# Genetic diversity and structure in *Leishmania infantum* populations from southeastern Europe revealed by microsatellite analysis

**DOI:** 10.1186/1756-3305-6-342

**Published:** 2013-12-05

**Authors:** Evi Gouzelou, Christos Haralambous, Maria Antoniou, Vasiliki Christodoulou, Franjo Martinković, Tatjana Živičnjak, Despina Smirlis, Francine Pratlong, Jean-Pierre Dedet, Yusuf Özbel, Seray Özensoy Toz, Wolfgang Presber, Gabriele Schönian, Ketty Soteriadou

**Affiliations:** 1 Laboratory of Molecular Parasitology, Hellenic Pasteur Institute, 127 Vasilissis Sofias Avenus, 11521, Athens, Greece; 2 Laboratory of Clinical Bacteriology, Parasitology, Zoonoses and Geographical Medicine, Faculty of Medicine, University of Crete, Heraklion, 710 03, Crete, Greece; 3 Veterinary Services of Cyprus, 1417, Nicosia, Cyprus; 4 Department for Parasitology and Parasitic Diseases with Clinics, Faculty of Veterinary Medicine, University of Zagreb, Heinzelova 55, 10000, Zagreb, Croatia; 5 Laboratoire de Parasitologie and Centre National de Référence des Leishmania, Université Montpellier 1 and CHU Montpellier, 39, Avenue Charles Flahault, 34295, Montpellier, France; 6 Department of Parasitology, Ege University Medical School, Bornova, Izmir, 35100, Turkey; 7 Institute of Microbiology and Hygiene, Charité University Medicine Berlin, Hindenburgdamm 30, D-12203, Berlin, Germany

## Abstract

**Background:**

The dynamic re-emergence of visceral leishmaniasis (VL) in south Europe and the northward shift to *Leishmania*-free European countries are well-documented. However, the epidemiology of VL due to *Leishmania infantum* in southeastern (SE) Europe and the Balkans is inadequately examined. Herein, we aim to re-evaluate and compare the population structure of *L. infantum* in SE and southwestern (SW) Europe.

**Methods:**

*Leishmania* strains collected from humans and canines in Turkey, Cyprus, Bulgaria, Greece, Albania and Croatia, were characterized by the *K26*-PCR assay and multilocus enzyme electrophoresis (MLEE). Genetic diversity was assessed by multilocus microsatellite typing (MLMT) and MLM Types were analyzed by model- and distance- based algorithms to infer the population structure of 128 *L. infantum* strains.

**Results:**

*L. infantum* MON-1 was found predominant in SE Europe, whilst 16.8% of strains were MON-98. Distinct genetic populations revealed clear differentiation between SE and SW European strains. Interestingly, Cypriot canine isolates were genetically isolated and formed a monophyletic group, suggesting the constitution of a clonal MON-1 population circulating among dogs. In contrast, two highly heterogeneous populations enclosed all MON-1 and MON-98 strains from the other SE European countries. Structure sub-clustering, phylogenetic and Splitstree analysis also revealed two distinct Croatian subpopulations. A mosaic of evolutionary effects resulted in consecutive sub-structuring, which indicated substantial differentiation and gene flow among strains of both zymodemes.

**Conclusions:**

This is the first population genetic study of *L. infantum* in SE Europe and the Balkans. Our findings demonstrate the differentiation between SE and SW European strains; revealing the partition of Croatian strains between these populations and the genetic isolation of Cypriot strains. This mirrors the geographic position of Croatia located in central Europe and the natural isolation of the island of Cyprus. We have analysed the largest number of MON-98 strains so far. Our results indicate extensive gene flow, recombination and no differentiation between MON-1 and MON-98 zymodemes. No correlation either to host specificity or place and year of strain isolation was identified. Our findings may be associated with intensive host migration and common eco-epidemiological characteristics in these countries and give valuable insight into the dynamics of VL.

## Background

*Leishmania infantum* is the main causative agent of human visceral leishmaniasis (HVL) and canine visceral leishmaniasis (CanL) and also responsible for sporadic cases of cutaneous leishmaniasis (CL) across the Mediterranean basin [1,2]. The concurrent species *Leishmania tropica* extending rarely to Europe via Greece [1,3-5] and *Leishmania major* present in North Africa and Middle East, are also accountable for anthroponotic and zoonotic CL, correspondingly [1].

Based on multilocus enzyme electrophoresis (MLEE), the current reference method for characterizing and classifying *Leishmania* strains [6], *L. infantum* MON-1 is the predominant zymodeme in all Mediterranean countries [7-9], while the genetically close *L. infantum* zymodeme MON-98 has occasionally been reported in Turkey [10], Cyprus [11], Egypt [12,13], Greece [5,14,15] and Portugal [16].

Dogs constitute the main reservoir host of *L. infantum* MON-1 [9] and probably MON-98 [5], while cats could be a secondary domestic *L. infantum* MON-1 reservoir [17,18]. Feline leishmaniasis is reported in countries highly endemic for CanL [19] and cats were recently found capable of transmitting MON-1 parasites to a proven *L. infantum* vector, *Phlebotomus perniciosus*[17]. The large number of sub-clinically infected dogs is a major veterinary and public health problem in south Europe [1]. Although clinically affected dogs are more prone to infect sand fly vectors, those being sub-clinically infected and seronegative can also transmit *Leishmania* parasites to sand flies and, therefore, contribute to the parasites’ maintenance [20-24].

Further to this, the distribution range of CanL has surpassed the limits of south Europe and is spreading toward northern European countries, presently considered non-endemic. In Germany, there is an estimate of 20,000 infected dogs that were either imported from the Mediterranean basin or infected after travelling to this region [25,26]. Furthermore, the presence of a *L. infantum* vector (*P. perniciosus*) in Germany and Switzerland has been recently reported [27]. The northward spread of CanL is also apparent in northern Spain, where new foci of CanL and the presence of its vectors (*Phlebotomus ariasi* and *P. perniciosus*) have been reported. In Italy, canine incidence has reached 30.3% in West Liguria [28], and in France seroprevalence fluctuates between 3.2% in Alpes-Maritimes and 26.5% in Corsica [29].

Concurrently, a considerable increase in VL cases due to *L. infantum* MON-1 is documented in traditionally endemic southeastern (SE) European countries. Since the first reported HVL cases in the islands of Spetses (1879) and Hydra (1881) [30], leishmaniasis is endemic in most islands and coastal regions of Greece and constitutes a significant veterinary and public health concern. During 1994–2001, HVL has re-emerged in inland regions of Greece, where seroprevalence reached 33.4% in Attica and 12.6% in Epirus [31]. During the same 8-year survey, the highest CanL seroprevalence in clinically affected dogs was found in Attica (48.4%) followed by Epirus (45.4%) and central Macedonia (40%) [31]. Of additional concern are the high seropositivity rates found in clinically healthy dogs across the mainland of Greece. According to recent studies, seroprevalence in healthy dog populations ranged between 22.4-30.1% in Attica, 21–24.4% in Epirus, 12.3%-17% in central Greece and 10.8%-20.5% in central Macedonia [31,32]. Among all Greek islands most dramatic changes have been witnessed in Crete, where CanL increased from 0.3% to 19.8% during 1990–2009 and HVL prevalence from 0 to 1.33 per 100,000 inhabitants [4,5].

In south Cyprus, HVL and CanL do co-exist, but show different epidemiological patterns. In contrast to other endemic Mediterranean countries, in Cyprus two separate concurrent disease patterns involve CanL due to *L. infantum* MON-1 [11,33] and HVL due to *Leishmania donovani* MON-37 [34-36]. CanL was widespread by 1935 and constituted a major veterinary health problem until 1945 [33]. Since the completion of subsequent eradication campaigns against malaria and echinococcosis, CanL gradually re-emerged in coastal areas and is now widespread and highly endemic. In particular, the overall canine incidence has risen from 1.7% to 14.9%-19.6% [11,33]. Currently, the highest prevalence rates found in all prefectures of south Cyprus are 33.3% (Larnaca), 32.4% (Limassol), 30.8% (Paphos) and 20% (Nicosia), while 24.3% of the PCR positive and 9.2% of the seropositive dogs are subclinically infected [11]. HVL due to *L. infantum* MON-1 has been absent since 1987 [33] and there are no official reports of infected natives even in areas highly endemic for CanL in south Cyprus. Yet, an increased incidence of human leishmaniasis possibly due to *L. infantum* has been reported in the northern part of the island [11,37].

In Turkey, the presence of leishmaniasis was first documented in the early 19th century in Trabzon (eastern part of Black Sea region) [38]. Nowadays, CanL is widespread across the country (0.7%-33.3%) [10,39] and highly endemic along the Aegean and Mediterranean coasts (11–30.4%) [10]. Childhood VL is also well-established, rising in recent years [10] and currently highly endemic in Central Anatolia, the Aegean and Mediterranean Regions [38]. However, the most recent data from Denizli province (Aegean Region) showed that all children were seronegative whereas adult HVL had increased significantly [10]. As in neighboring Cyprus, parasites belonging to a genetically distinct *L. infantum* non MON-1 group have been reported to cause VL or CL in Turkey [34,36].

In Croatia, HVL and CanL existed across the Dalmatian coasts (Split, Makarska, and Dubrovnik areas) until 1957. The majority of reported HVL cases (90.4%) involved children under 10 years old and 4% of these cases were fatal [40]. The disease occurred only sporadically for 30 years due to mass spraying with insecticides for malaria control, but dramatically re-emerged during the war and postwar periods. During the past two decades, human incidence has reached 0.4/10^5^ of the population in southern Dalmatia [40] and is still considered a paediatric disease as 64% of cases involve children [41]. Reports on HVL cases suggest that existing differences regarding the clinical course of infection and/or disease outcome may be associated, amongst other factors, with variations of parasite species [42]. CanL is endemic in the city of Split (14.7%) and across the enzootic Split-Dalmatia County (7.1%-42.8%), while 45.7% of seropositive dogs were found to be sub-clinically infected [43].

In Albania, the incidence and geographical spread of HVL and CanL has gradually increased since World War II. However, the disease sharply re-emerged during 1997–2001 with an average 173 HVL cases/year and 2.8/10^4^ population morbidity, which exceeded 1/10^3^ lethality in active foci across north and central Albania [44]. Endemic zones are no longer restricted to northern regions or coastal and hilly areas. Children comprise 70–87.7% of HVL cases with an annual incidence of 25/10^5^ (0–6 year age group) [44-46]. Adult HVL cases and HIV/*Leishmania* co-infections are also recorded [45]. Since 1977, CanL is widespread across the country with 16-17% seroprevalence recorded in Tirana and several other districts [44,46].

In Bulgaria, canine seroprevalence has gradually decreased from 81% in 1941 to 13.8% in 1993 [47,48]. However, during 1988–2004 an HVL outbreak occurred in south Bulgaria and an unexpectedly high morbidity rate (50.7%) was noticed in Stara Zagora. In 2005, two autochthonous HVL cases were reported in the same region, but all stray dogs tested were found seronegative [49]. Conversely, in 2006 two breed dogs, which were not imported and had never travelled outside Bulgaria, were diagnosed with CanL [47]. *L. infantum* is incriminated for human and canine disease [49], underreporting is considered mild and the estimated annual VL incidence ranged between 8 and 12 cases/year during 2004–2008 [50].

The epidemiology of VL in these SE European and Balkan countries has been either inadequately or never examined so far. Multilocus microsatellite typing (MLMT), a powerful tool for phylogenetic and population genetic studies in *Leishmania*[51-55], previously showed that *L. infantum* MON-1 strains from Greece differentiate from those of Western Mediterranean [15] and North Africa [56,57]. In addition, there is no indication of subdivision between Turkish and Greek strains or within Greek strains as well as no differentiation between MON-1 and MON-98 strains from Greece [15]. These findings prompted us to initiate a comprehensive population genetic study in SE Europe. For this, 109 *Leishmania* strains (25 human and 84 canine isolates) were collected from Turkey, Cyprus, Bulgaria, Greece, Albania and Croatia, and characterized by MLEE and *K26* typing. The analysis of *L. infantum* strains by MLEE has demonstrated a broad enzymatic polymorphism within this species but has limited epidemiological applicability in Europe due to the predominance of MON-1 [6]. The *K26*-PCR assay, which is specific for the *L. donovani* complex, allows simultaneous discrimination between *L. infantum* MON-1 and more than nine *L. infantum* non MON-1 zymodemes that are known to exist in Europe [14,58,59]. Notably, the 626 bp *K26* amplicon corresponds only to zymodeme MON-1, while the 940 bp *K26* amplicon may correspond either to zymodeme MON-1 or MON-98. For microsatellite typing, a standard set of highly discriminative markers [52] was employed for all strains from SE and their MLMT profiles were compared to 19 previously analyzed strains of *L. infantum* MON-1 from southwestern (SW) European countries (France, Spain and Portugal) aiming to re-assess the genetic diversity and population structure of *L. infantum* in SE Europe.

## Methods

### Leishmania strains and study area

Table 1 summarizes the characteristics of the 128 *L. infantum* strains included in the study. Fifty-four strains were isolated from 20 human and 34 canine VL cases in diverse geographic regions of the mainland and islands of Greece. Sixteen human and canine isolates were obtained from Turkey, mainly from the Aegean region. Of these, nine MON-1 or MON-98 strains from Greece and two Turkish strains of unknown zymodeme type (strains EP3 and EP16) were previously analyzed in different studies [15,52]. Twenty-three canine isolates were collected from five prefectures of southern Cyprus and sixteen strains were isolated in Bulgaria, Albania and Croatia, mostly from canines. Nineteen previously analyzed *L. infantum* MON-1 strains from France, Spain and Portugal [15,52] were incorporated for comparison.

**Table 1:** **Characteristics of the 128** ***Leishmania infantum *strains studied**

**Origin**	**Strains**	**WHO code**	**Periphery/Region-Prefecture/Province (District)**	**Pathology**^**a**^	**MON**^**b**^	***K26 *** **amplicon**^**b**^	**MLMT profile**^**c**^	**Structure K = 4**^**d**^
TR(16)	EP3*	MHOM/TR/1994/EP3	Aegean-Izmir	VL	n.d.	n.d.	35	pop2
	EP16*	MCAN/TR/1996/EP16	Aegean-Manisa	CanL	n.d.	n.d.	36	pop2
	EP43	MCAN/TR/2000/EP43	Aegean-Aydin(Kuşadası)	CanL	98	870/940	*37*	pop4 (sub4A)
	EP44	MCAN/TR/2000/EP44	Aegean-Aydin(Kuşadası)	CanL	98	870/940	*37*	pop4 (sub4A)
	EP49	MCAN/TR/2000/EP49	Aegean-Aydin(Kuşadası)	CanL	98	870/940	*37*	pop4 (sub4A)
	EP50	MCAN/TR/2000/EP50	Aegean-Aydin(Kuşadası)	CanL	98	870/940	38	pop4 (sub4A)
	EP53	MCAN/TR/2000/EP53	Aegean-Aydin(Kuşadası)	CanL	98	870/940	39	pop4 (sub4A)
	EP55	MCAN/TR/2000/EP55	Aegean-Aydin(Kuşadası)	CanL	98	870/940	40	pop4 (sub4A)
	EP56	MCAN/TR/2000/EP56	Marmara-Bilecik	CanL	1	870/940	*25*	pop2
	EP57	MCAN/TR/2001/EP57	Aegean-Muğla	CanL	n.d.	870/940	*25*	pop2
	EP58	MCAN/TR/2001/EP58	Aegean-Muğla	CanL	n.d.	940	*41*	pop2
	EP68	MCAN/TR/2001/EP68	Aegean-Aydin(Kuşadası)	CanL	n.d.	940	*41*	pop2
	EP88	MCAN/TR/2003/EP88	Black Sea-Çorum	CanL	1	626	42	pop3 (sub3B^1^)
	EP89	MHOM/TR/2003/EP89	Aegean-Manisa(Alaşehir)	VL	n.d.	870/940	43	pop2
	EP126	MCAN/TR/2005/EP126	Aegean -Muğla(Bodrum)	CanL	1	870/940	*13*	pop2
	EP134	MCAN/TR/2007/EP134	Mediterranean-Hatay(Antakya)	CanL	1	626	44	pop4 (sub4C^1^)
CY(23)	CD1	MCAN/CY/2001/CD1	Limassol	CanL	1	626	*29*	pop1 (sub1A)
	CD13	MCAN/CY/2003/CD13	Limassol	CanL	1	626	*29*	pop1 (sub1A)
	CD21	MCAN/CY/2004/CD21	Paphos	CanL	1	626	*29*	pop1 (sub1A)
	CD22	MCAN/CY/2004/CD22	Limassol	CanL	1	626	*29*	pop1 (sub1A)
	CD34	MCAN/CY/2005/CD34	Limassol	CanL	1	626	30	pop1 (sub1B)
	CD37	MCAN/CY/2005/CD37	Paphos	CanL	1	626	*29*	pop1 (sub1A)
	CD40	MCAN/CY/2005/CD40	Paphos	CanL	1	626	*29*	pop1 (sub1A)
	CD43	MCAN/CY/2005/CD43	Paphos	CanL	1	626	*29*	pop1 (sub1A)
	CD44 cl.5^e^	MCAN/CY/2005/CD44cl.5	Paphos	CanL	1	626	31	pop1 (sub1B)
	CD48	MCAN/CY/2005/CD48	Limassol	CanL	1	626	*29*	pop1 (sub1A)
	CD51	MCAN/CY/2005/CD51	Paphos	CanL	1	626	*29*	pop1 (sub1A)
	CD57	MCAN/CY/2005/CD57	Nicosia	CanL	1	626	*29*	pop1 (sub1A)
	CD62	MCAN/CY/2005/CD62	Paphos	CanL	1	626	*29*	pop1 (sub1A)
	CD68	MCAN/CY/2005/CD68	Paphos	CanL	1	626	*29*	pop1 (sub1A)
	CD74	MCAN/CY/2006/CD74	Famagusta	CanL	1	626	*29*	pop1 (sub1A)
	CD63	MCAN/CY/2005/CD63	Limassol	CanL	1	626	*29*	pop1 (sub1A)
	CD66	MCAN/CY/2005/CD66	Nicosia	CanL	1	626	*29*	pop1 (sub1A)
	CD85	MCAN/CY/2006/CD85	Nicosia	CanL	1	626	32	pop1 (sub1B)
	CD87	MCAN/CY/2006/CD87	Paphos	CanL	1	626	*29*	pop1 (sub1A)
	CD88	MCAN/CY/2006/CD88	Paphos	CanL	1	626	*33*	pop1 (sub1B)
	CD90	MCAN/CY/2006/CD90	Paphos	CanL	1	626	*29*	pop1 (sub1A)
	CD91	MCAN/CY/2006/CD91	Paphos	CanL	1	626	*33*	pop1 (sub1B)
	CD108	MCAN/CY/2006/CD108	Larnaca	CanL	1	626	34	pop1 (sub1B)
BG(1)	BG1	MCAN/BG/2008/BG1	Blagoevgrad	CanL	1	940	*11*	pop2
GR(54)	GH2*	MHOM/GR/2001/GH2	Attica-Athens	VL	1	870/940	1	pop2
	GH5*	MHOM/GR/2001/GH5	Crete-Lasithi/Ag.Nikolaos	VL	1	870/940	*2*	pop2
	GH8*	MHOM/GR/2001/GH8	Attica-Athens	VL	1	870/940	*3*	pop2
	GH9*	MHOM/GR/2001/GH9	Attica-Athens	VL	1	870/940	4	pop4 (sub4C^1^)
	GH10*	MHOM/GR/2001/GH10	Attica-Athens	VL	1	870/940	*2*	pop2
	GH11*	MHOM/GR/2001/GH11	Attica-Athens	VL	1	870/940	*3*	pop2
	GH13	MHOM/GR/2003/GH13	Crete-Heraklion	VL	98	870/940	*5*	pop4 (sub4A)
	GH21	MHOM/GR/2004/GH21	Crete-Heraklion	VL	1	870/940	6	pop2
	GH22	MHOM/GR/2004/GH22	Crete-Rethymno	VL	98	870/940	*5*	pop4 (sub4A)
	GH23	MHOM/GR/2004/GH23	Crete-Rethymno	VL	98	870/940	7	pop4 (sub4C^1^)
	GH24	MHOM/GR/2004/GH24	Attica-Athens	VL	1	626	8	pop4 (sub4C^2^)
	GH26	MHOM/GR/2004/GH26	Crete-Lasithi/Sitia	VL	1	870/940	9	pop2
	GH27	MHOM/GR/2005/GH27	Crete-Heraklion	VL	1	940	10	pop2
	LEM2289	MHOM/GR/1990/LA1037	Attica-Athens	VL	1	870/940	*11*	pop2
	PAPADO	MHOM/GR/1980/PAPADO	Attica-Athens	VL	1	870/940	*3*	pop2
	GH49	MHOM/GR/2008/GH49	North Aegean Sea-Lesbos/Mytilene	VL	1	870/940	*2*	pop2
	GH51	MHOM/GR/2008/GH51	Central Greece-Euboea/Halkida	VL	1	940	12	pop2
	GH54	MHOM/GR/2009/GH54	Central Greece-Phthiotis(Lamia)	VL	1	870/940	*13*	pop2
	GH55	MHOM/GR/2009/GH55	Crete-Heraklion	VL	1	870/940	14	pop2
	GH56	MHOM/GR/2009/GH56	Peloponnese-Arcadia (Tripoli)	VL	1	870/940	15	pop2
	GD3*	MCAN/GR/2001/GD3	Crete-Heraklion	CanL	98	870/940	16	pop4 (sub4C^1^)
	GD4*	MCAN/GR/2001/GD4	Crete-Heraklion	CanL	98	870/940	17	pop4 (sub4C^1^)
	GD9	MCAN/GR/2002/GD9	Crete-Heraklion	CanL	1	870/940	18	pop4 (sub4C^2^)
	GD12	MCAN/GR/2003/GD12	Crete-Lasithi/Sitia	CanL	1	870/940	*2*	pop2
	GD14	MCAN/GR/2003/GD14	Crete-Heraklion	CanL	98	870/940	*3*	pop2
	GD17	MCAN/GR/2004/GD17	Crete-Heraklion	CanL	98	870/940	*5*	pop4 (sub4A)
	GD18	MCAN/GR/2004/GD18	Crete-Heraklion	CanL	1	626	*19*	pop4 (sub4C^2^)
	GD19	MCAN/GR/2004/GD19	Crete-Heraklion	CanL	1	626	*19*	pop4 (sub4C^2^)
	GD20	MCAN/GR/2004/GD20	Crete-Heraklion	CanL	1	870/940	*11*	pop2
	GD23	MCAN/GR/2004/GD23	Crete-Lasithi/Sitia	CanL	1	940	*2*	pop2
	GD24	MCAN/GR/2004/GD24	Crete-Lasithi/Sitia	CanL	1	870/940	*2*	pop2
	GD25	MCAN/GR/2004/GD25	Crete-Lasithi/Sitia	CanL	1	940	*2*	pop2
	GD26	MCAN/GR/2004/GD26	Crete-Lasithi/Sitia	CanL	1	870/940	*2*	pop2
	GD31	MCAN/GR/2005/GD31	Crete-Lasithi/Sitia	CanL	1	870/940	*2*	pop2
	GD32	MCAN/GR/2005/GD32	Crete-Lasithi/Sitia	CanL	1	940	*2*	pop2
	GD33	MCAN/GR/2005/GD33	Crete-Lasithi/Sitia	CanL	1	870/940	*2*	pop2
	GD35	MCAN/GR/2005/GD35	Crete-Lasithi/Sitia	CanL	98	870/940	*5*	pop4 (sub4A)
	GD36	MCAN/GR/2005/GD36	Crete-Lasithi/Sitia	CanL	98	870/940	*5*	pop4 (sub4A)
	GD71	MCAN/GR/2006/GD71	Crete-Lasithi	CanL	98	870/940	*3*	pop2
	GD102	MCAN/GR/2006/GD102	Attica (Rafina)	CanL	98	870/940	*3*	pop2
	GD104	MCAN/GR/2006/GD104	Crete-Heraklion	CanL	98	870/940	20	pop4 (sub4A)
	GD112	MCAN/GR/2006/GD112	Crete-Heraklion	CanL	98	870/940	21	pop4 (sub4A)
	GD154	MCAN/GR/2008/GD154	Central Macedonia-Thessaloniki	CanL	1	940	*22*	pop2
	GD167	MCAN/GR/2008/GD167	Thessaly-Trikala	CanL	98	870/940	*11*	pop2
	GD192	MCAN/GR/2008/GD192	South Aegean-Dodecanese/Rhodes	CanL	1	626/870/940	*22*	pop2
	GD201	MCAN/GR/2008/GD201	East Macedonia-Drama	CanL	1	870/940	23	pop2
	GD204	MCAN/GR/2009/GD204	East Macedonia-Kavala	CanL	1	870/940	24	pop2
	GD210	MCAN/GR/2009/GD210	Epirus-Ioannina/Dodoni	CanL	1	626	*25*	pop2
	GD211	MCAN/GR/2009/GD211	Peloponnese-Argolis	CanL	98	870/940	*3*	pop2
	GD220	MCAN/GR/2009/GD220	West Greece-Achaea	CanL	1	940	26	pop2
	GD221	MCAN/GR/2009/GD221	Epirus-Arta	CanL	1	626	*25*	pop2
	GD223	MCAN/GR/2009/GD223	Ionian Islands-Corfu(Kerkyra)	CanL	1	626/940	27	pop4 (sub4C^2^)
	GD229	MCAN/GR/2009/GD229	South Aegean-Cyclades	CanL	1	940	*13*	pop2
	GD237	MCAN/GR/2010/GD237	Central Macedonia-Imathia	CanL	1	940	28	pop2
AL(5)	AH2	MHOM/AL/2006/AH2	unknown	VL	1	870/940	52	pop2
	AH4	MHOM/AL/2006/AH4	unknown	VL	1	870/940	*11*	pop2
	LEM3962	MHOM/AL/2000/516LEZH	Tirana	VL	1	870/940	53	pop4 (sub4B)
	AD16	MCAN/AL/2004/AD16	unknown	CanL	1	n.d.	*54*	pop2
	LEM3894	MCAN/AL/98/C78L574	Tirana	CanL	1	870/940	*54*	pop2
HR(10)	Lu	MCAN/HR/2009/Lu	Unknown	CanL	1	626	*45*	pop3 (sub3B^2^)
	Ne	MCAN/HR/2009/Ne	Cetina(Brač)	CanL	1	626	46	pop3 (sub3B^2^)
	Me	MCAN/HR/2009/Me	Cetina(Makarska)	CanL	1	626	*47*	pop3 (sub3B^2^)
	Mr	MCAN/HR/2008/Mr	unknown	CanL	1	626	*48*	pop4 (sub4B)
	GS	MCAN/HR/2009/GS	unknown	CanL	1	626	49	pop4 (sub4B)
	Ć	MCAN/HR/2009/Ć	unknown	CanL	1	626	*47*	pop3 (sub3B^2^)
	U	MCAN/HR/2009/U	Dubrava(Korčula)	CanL	1	626	*48*	pop4 (sub4B)
	Pa	MCAN/HR/2009/Pa	Dubrava(Korčula)	CanL	1	626	50	pop4 (sub4B)
	SO	MCAN/HR/2011/SO	Dubrovnik	CanL	1	626	*45*	pop3 (sub3B^2^)
	AL	MCAN/HR/2011/AL	Split	CanL	1	626	51	pop3 (sub3B^1^)
FR(3)^R^	LG1*	MHOM/FR/1996/LEM75	Cévennes	VL	1	626	69	pop3 (sub3B^1^)
	LG2*	MHOM/FR/1995/LPN114	Côte d’Azur	VL	1	626	70	pop3 (sub3B^1^)
	LG4*	MHOM/FR/1997/LSL29	Cévennes	CL	1	626	71	pop3 (sub3B^1^)
ES(11)^R^	LLM-1139*	MCAN/ES/2002/LLM-1139	Ibiza	CanL	1	626	55	pop3 (sub3A)
	LLM-1228*	MCAN/ES/2003/LLM-1228	Ibiza	CanL	1	626	56	pop3 (sub3A)
	LLM-1241*	MCAN/ES/2003/LLM-1241	Ibiza	CanL	1	626	57	pop3 (sub3A)
	PMI*	MHOM/ES/1993/PM1	Majorca	VL	1	626	58	pop3 (sub3A)
	LLM-981*	MHOM/ES/2001/LLM-981	Majorca	VL	1	626	59	pop3 (sub3A)
	LLM-1008*	MCAN/ES/2001/LLM-1008	Majorca	CanL	1	626	60	pop3 (sub3A)
	LLM-1150*	MHOM/ES/2002/LLM-1150	Majorca	CL(HIV+)	1	626	61	pop3 (sub3A)
	LLM-1038*	MCAN/ES/2001/LLM-1038	Majorca	CanL	1	626	62	pop3 (sub3B^1^)
	LLM-1116*	MCAN/ES/2002/LLM-1116	Madrid	CanL	1	626	63	pop3 (sub3B^1^)
	LLM-1136*	MCAN/ES/2001/LLM-1136	Madrid	CanL	1	626	64	pop3 (sub3B^1^)
	LLM-1181*	MHOM/ES/2002/LLM-1181	Madrid	VL(HIV+)	1	626	*65*	pop3 (sub3B^1^)
PT(5)^R^	IMT184*	MHOM/PT/1993/IMT184	Lisbon	VL	1	626	*65*	pop3 (sub3B^1^)
	ΙΜΤ339*	MCAN/PT/2003/IMT339	Lisbon	CanL	1	626	66	pop3 (sub3B^1^)
	IMT205*	MCAN/PT/1995/IMT205	Alentejo	CanL	1	626	*65*	pop3 (sub3B^1^)
	IMT288*	MHOM/PT/2002/IMT288	Alto Douro	VL	1	626	67	pop3 (sub3B^1^)
	ΙΜΤ316*	MCAN/PT/2003/IMT316	Lisbon	CanL	1	626	68	pop3 (sub3B^1^)

^R^reference strains included for comparison, the number of strains per geographical source is given in parenthesis; *strains analyzed in previous studies [14,15,36,52,55,61,68]; TR, Turkey; CY, Cyprus; BG, Bulgaria; GR, Greece; AL, Albania; HR, Croatia; FR, France; ES, Spain; PT, Portugal; ^a^VL, visceral leishmaniasis; CanL, canine leishmaniasis; CL (HIV+), cutaneous leishmaniasis (co-infection with HIV); ^b^based on MLEE and *K26* typing; n.d. not done; ^c^The 71 microsatellite profiles shared between different strains are indicated in italics; ^d^Population assignment at K = 4 by STRUCTURE analysis, sub-structuring upon re-analysis is shown in parenthesis; ^e^*L. infantum* MON-1 clone isolated from a case of mixed infection with MON-1 and MON-37 strains [34,36].

### Parasite cultures, cloning and DNA extraction

*Leishmania* promastigotes were cultured at 25°C in RPMI 1640 or M199 cell growth medium supplemented with 10-20% heat-inactivated FBS, 5U/ml penicillin, 5 μg/ml streptomycin and 10 mM HEPES buffer. The number of *in vitro* passages was limited to a minimum to avoid contamination and genetic drift due to serial sub-culturing. Clonal populations from the mixed *L. infantum* MON-1/*L. donovani* MON-37 culture of the CD44 strain were obtained as described elsewhere [36]. *Leishmania* DNA was extracted from parasite cultures using a classical phenol/chloroform protocol [60] or the standard protocol for cultured cells using the NucleoSpin^®^ Tissue kit (Macherey-Nagel). Extracted DNA was respectively dissolved in dH_2_O or eluted in TE buffer and stored at -20°C, whereas working solutions were kept at 4°C until use.

### K26-PCR, MLEE and MLMT analysis

The *K26*-PCR assay was applied to discriminate MON-1 from other *L. infantum* zymodemes according to the length polymorphism of the *K26* gene, as described previously [14]. MLEE analysis based on starch gel electrophoresis of fifteen enzymatic loci [6] was performed for all but six Turkish strains, at the University of Montpellier (France), to complement *K26* typing.

Multilocus microsatellite typing (MLMT) was applied using fourteen microsatellite markers previously found highly discriminative within the *L. donovani* complex [52,61]. Microsatellite fragments were amplified using established PCR protocols [52]. Amplicons were resolved on high-resolution MetaPhor^®^ gels (3.5-4.5%) using a large gel horizontal electrophoresis system with built-in recirculation (Owl Large Gel System, Thermo Scientific) and screened for length polymorphisms using the AlphaImager^®^ imaging system (Alpha Innotech Corp.) as previously described [36]. Alternatively, PCR assays were performed using fluorescence conjugated primers (WellRed dyes, Proligo) and analyzed by capillary electrophoresis (CEQ™ 8000, Beckman Coultier), as described elsewhere [36]. Control strains were included in both PCR protocols and reading methods to confirm the reproducibility of the results.

### Population structure and phylogenetic analysis

The MLMT dataset of 128 strains, including that of 30 previously analyzed strains [15,36,52,55,61], was processed by models based on Bayesian statistics and genetic distances. Model-based analysis was performed by STRUCTURE v2.3.1 [62] to infer the population structure and identify genetically distinct *L. infantum* clusters. Based on multilocus genotypes and independent of a mutation model, this algorithm was used to determine patterns of allele frequencies per analyzed locus and estimated the most probable degree of differentiation. Individuals were assigned in discrete genetic populations and exposed their membership to each estimated population by genotypic fractions. The allele frequencies among populations were correlated by admixture modeling for a series of runs using a ‘burn-in’ period of 2x10^5^ iterations and probability estimates based on 2×10^6^ of MCMC repeats. For each possible number of populations between K = 1 and K = 10, ten independent simulations were conducted to estimate the delta (Δ)K values [63]. The likelihood of population number was calculated by STRUCTURE HARVESTER [64], implementing the Evanno method [63]. Subsequent STRUCTURE analyses were performed to achieve assignment at different hierarchies.

Phylogenetic analysis was performed based on microsatellite genetic distances, which were calculated by MICROSAT software using the proportion of shared alleles distance measure (D_AS_). The resulting distance matrix input was processed by PHYLIP v3.6 to construct a Neighbour-joining (NJ) tree with confidence intervals by bootstrapping (1000 replications). A consensus tree with the corresponding bootstrap values and branch lengths was re-constructed using GENEIOUS v5.4 [65] and the derived mid-point rooted NJ tree was edited by FIGTREE (http://tree.bio.ed.ac.uk/software/figtree). Genetic relatedness and recombination were evaluated through NeighborNet networks constructed by SplitsTree4 software and through the population membership coefficients estimated by STRUCTURE. The MLMT data were analyzed by GDA (http://hydrodictyon.eeb.uconn.edu/people/plewis/software.php) with respect to the proportion of polymorphic loci (P), allelic variation (A), inbreeding coefficient (F_*IS*_), expected (H_*e*_) and observed (H_*o*_) heterozygosity. GDA analysis was performed per microsatellite locus and per population/subpopulation defined by STRUCTURE. *F*-statistics were estimated for all populations/subpopulations to evaluate genetic differentiation and gene flow. For this, the F_*ST*_ values (pairwise Wright’s fixation index) with their corresponding *p*-values (confidence test) were calculated using MSA [66]. Moreover, the total strain-set was sorted by geographical source to calculate the descriptive statistics per endemic country. Similarly, a separate descriptive analysis was performed for Turkish/Greek MON-1 and MON-98 strains based on their zymodeme type and origin.

The spatial distribution of the observed genetic structure at population and individual level was assessed based on allelic similarity by factorial correspondence analysis (FCA) using GENETIX v4.03 [67].

### Ethics statement

The *Leishmania* strains designated with an asterisk (*, Table 1) and those originating from SW Europe (^R^, Table 1), which were used in this microsatellite analysis for comparison, have been the object of previous publications [14,15,36,52,55,61,68].

Strains from Albania were obtained from the National Reference Centre of *Leishmania* at Montpellier, France. Ethical considerations are described in Petrela *et al.*[45]. Strains from Greece were obtained from the already-existing “*Leishmania* cryobank collection” of the Medical School of the University of Crete. Informed written consent was obtained from all patients. Both the study and protocols used were approved by the Ethical Scientific Committee of the Medical School (University of Crete, Greece). Ethical considerations are described in Kuhls *et al*. [14,15,36,52,55,61,68]. Strains from Turkey were collected in the context of previous studies, as indicated above. Human strains were obtained from an already-existing “*Leishmania* Group cryobank collection”. Informed written consent was obtained from all patients. The study was approved by Local Animal Care and Ethics Committee of the School of Medicine Izmir, Turkey.

All samples were coded and anonymized, and all *Leishmania* strains were isolated as part of normal diagnosis. Dog samples were collected by veterinarians after the owner’s consent, following protocols that were approved by the Ethics Committees of the respective Institutions in Turkey, Cyprus, Greece and Croatia. Questionnaires with personal, epidemiological, and clinical data for each dog were also completed.

Approval from the respective Institutions to use the samples for research purposes was received in all cases. The present study was also reviewed and approved by the Ethics Committee of the Hellenic Pasteur Institute, Athens, Greece.

## Results

### K26-PCR and MLEE analysis

**Figure 1:**
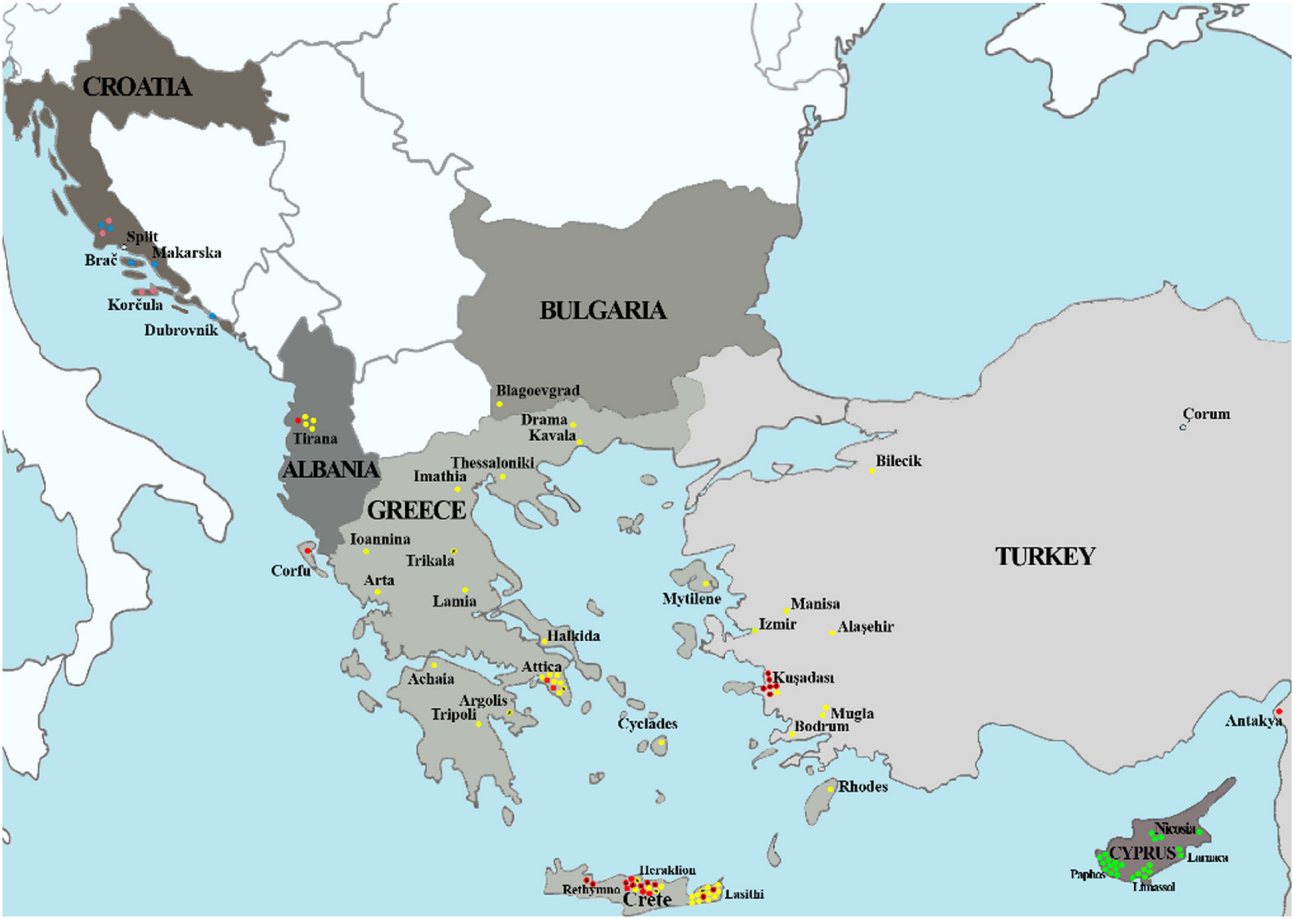
**Geographical distribution of the 128** ***L. infantum *strains studied.** Strains are represented by coloured circles that correspond to their population assignment at K = 4 by STRUCTURE analysis (Figure 2A); Population 1-green, population 2-yellow, population 3-blue and population 4-red circles. The circles representing the Turkish strain isolated from Black Sea region (Çorum prefecture) and all strains from Croatia are colored according to their subpopulation assignment as in Figure 4. *L. infantum* MON-98 strains are indicated with an asterisk. Strains included for comparison from France, Spain and Portugal (Table 1) are not shown.

Of the total 128 strain-set, 125 strains were characterized at species level and zymodeme type by *K*26-PCR, and 122 of them also by MLEE, in the context of either this or previous studies [15,36] (Table 1**)**. A single 626 bp *K26* amplicon characteristic for *L. infantum* MON-1 was presented by a few Turkish and Greek strains as well as by all strains from Cyprus and Croatia, including two clones of strain CD44 (CD44cl.4 and CD44cl.5) isolated from a canine with a mixed *L. infantum* MON-1/*L. donovani* MON-37 infection in Cyprus [34,36]. The 940 bp *K26* amplicon, which could correspond to either *L. infantum* MON-1 or MON-98 zymodeme and was observed so far only in Greek strains, was presented by all strains from Turkey, Bulgaria and Albania, except for two Turkish strains from regions other than the Aegean. These were strains EP88 and EP134 isolated at the Black Sea and the Mediterranean region close to Cyprus, respectively. Besides the main 940 bp *K26* amplicon observed for most strains from Greece, two Greek strains (GD192 and GD223) gave multiple *K26*-PCR products (626/870/940 bp and 626/940 bp, respectively). In addition to seven previously typed *L. infantum* MON-98 strains from Crete, MLEE analysis identified 14 new strains of MON-98 from eight human and canine isolates from mainland regions of Greece and the island of Crete as well as six canine isolates from the Aegean region of Turkey (Kuşadasi) (Figure 1). Overall, our sample-set comprises 101 MON-1 strains, 21 MON-98 strains from Turkey and Greece, and 6 Turkish *L. infantum* strains of unknown zymodeme type. Interestingly, 21 out of 70 Turkish and Greek strains (30%) were identified as MON-98, which is the highest percentage of this zymodeme found so far in Turkey and Greece.

### Analysis of microsatellite loci and correlation with zymodeme type

The inter- and intra-zymodeme genetic diversity among 128 *L. infantum* MON-1 and MON-98 strains from south Europe was assessed by a panel of previously described microsatellite markers located on nine different chromosomes [15,36,52,53,61]. Table 2 summarizes the characteristics and descriptive statistics per analyzed locus for the overall strain-set.

**Table 2:** **Characteristics and descriptive statistics by microsatellite locus for the 128** ***L. infantum *strains analysed in this study**

**Locus**	**Chr**	**Repeat array**	**P**	**A**	**H**_***e***_	**H**_***o***_	**F**_***IS***_
Lm2TG	1	TG (22–27)	1	6	0.69	0.09	0.88
Lm4TA	1	TA (10–17)	1	8	0.76	0.10	0.87
Li41-56 (B)	36	CA (9–11)	1	3	0.37	0.00	1.00
Li71-7 (R)	30	CA (12–14)	1	3	0.12	0.02	0.87
Li22-35 (E)	1	CA (10–13,15,16,18)	1	7	0.64	0.05	0.92
Li23-41 (F)	25	GT (13,15-17)	1	4	0.35	0.02	0.96
Li45-24 (G)	36	CA (14–18)	1	5	0.51	0.02	0.95
CS20	19	TG (18–20)	1	3	0.29	0.01	0.97
LIST7031	10	CA (11,12)	1	2	0.11	0.01	0.93
LIST7039	30	CA (14–16)	1	3	0.09	0.01	0.92
Li46-67 (C)	31	CA (9)	0	1	0.00	0.00	0.00
Li71-33 (P)	31	TG (10–12)	1	3	0.03	0.00	1.00
Li71-5/2 (Q)	35	CA (8–10)	1	3	0.08	0.00	1.00
TubCA	34	CA (9)	0	2	0.01	0.00	1.00
**Overall**			**0.86**	**3.79**	**0.29**	**0.02**	**0.92**

Locus, analysed microsatellite marker; Chr, chromosome location; Repeat array per locus, in parenthesis are the overall dinucleotide repeat numbers identified; P, proportion of polymorphic loci; A, number of alleles per locus; H_*e*_, Nei’s unbiased expected heterozygosity; H_*o*_, observed heterozygosity; F_*IS*_, inbreeding coefficient.

Different degrees of polymorphism were observed among the analyzed loci. As shown in Table 2, the highest allelic richness, expected and observed heterozygosities were demonstrated for markers Lm4TA, Lm2TG and Li22-35 (E), with the expected heterozygosity (mean H_*e*_ = 0.29) always being much higher than the observed (mean H_*o*_ = 0.02). Inbreeding coefficients (F_*IS*_) were substantially high (mean F_*IS*_ = 0.92), as four markers presented absolute heterozygote deficiency (F_*IS*_ = 1), and marker Li46-67 (C) was monomorphic as previously observed [52,54].

Separate analysis was performed to investigate intra-zymodeme diversity and avoid possibly biased results due to the larger number of MON-1 strains in the total sample-set. The measures of genetic variability of Turkish/Greek strains demonstrated extensive inbreeding in both zymodeme populations and just trivial differences between MON-1 and MON-98 zymodemes (Table 3). However, some alleles at markers Lm2TG (22 repeats), CS20 (22 repeats), Li45-24 (G) (18 repeats) and LIST7039 (16 repeats) were only found in MON-98 strains.

**Table 3:** Genetic diversity between and within MON-1 and MON-98 strains from Greece and Turkey based on the MLMT profiles in 14 analyzed markers

**Population**	**n**	**Matches**	**P**	**A**	**H**_***e***_	**H**_***o***_	**F**_***IS***_
TR/GR MON-1	44	2	0.71	2.64	0.19	0.02	0.87
GR MON-1	40	3 (5)	0.57	2.14	0.17	0.02	0.86
TR MON-1	4	0	0.64	1.93	0.24	0.03	0.88
TR/GR MON-98	21	0	0.57	2.21	0.15	0.03	0.82
GR MON-98	15	1	0.43	1.93	0.17	0.02	0.89
TR MON-98	6	1	0.29	1.29	0.05	0.05	0.00

Nine identical genotypes (matches) were identified between MON-1 and MON-98 strains from Turkey (TR) and Greece (GR). The number in parenthesis includes two common genetic profiles found between MON-1 and MON-98 strains from Greece. n, number of analyzed strains per population; P, proportion of polymorphic loci; A, number of alleles per locus; H_*e*_, Nei’s unbiased expected heterozygosity; H_*o*_, observed heterozygosity; F_*IS*_, inbreeding coefficient.

### Population structure inference

The Bayesian model-based clustering algorithm implemented in the STRUCTURE software was run by increasing K values from K = 2 to K = 10 and with seven geographically-defined strain-groups, as strains from France (FR), Spain (ES) and Portugal (PT) (Table 1) representing SW Europe were considered to constitute one population, according to earlier reports [15,36,52,55,61].

**Figure 2:**
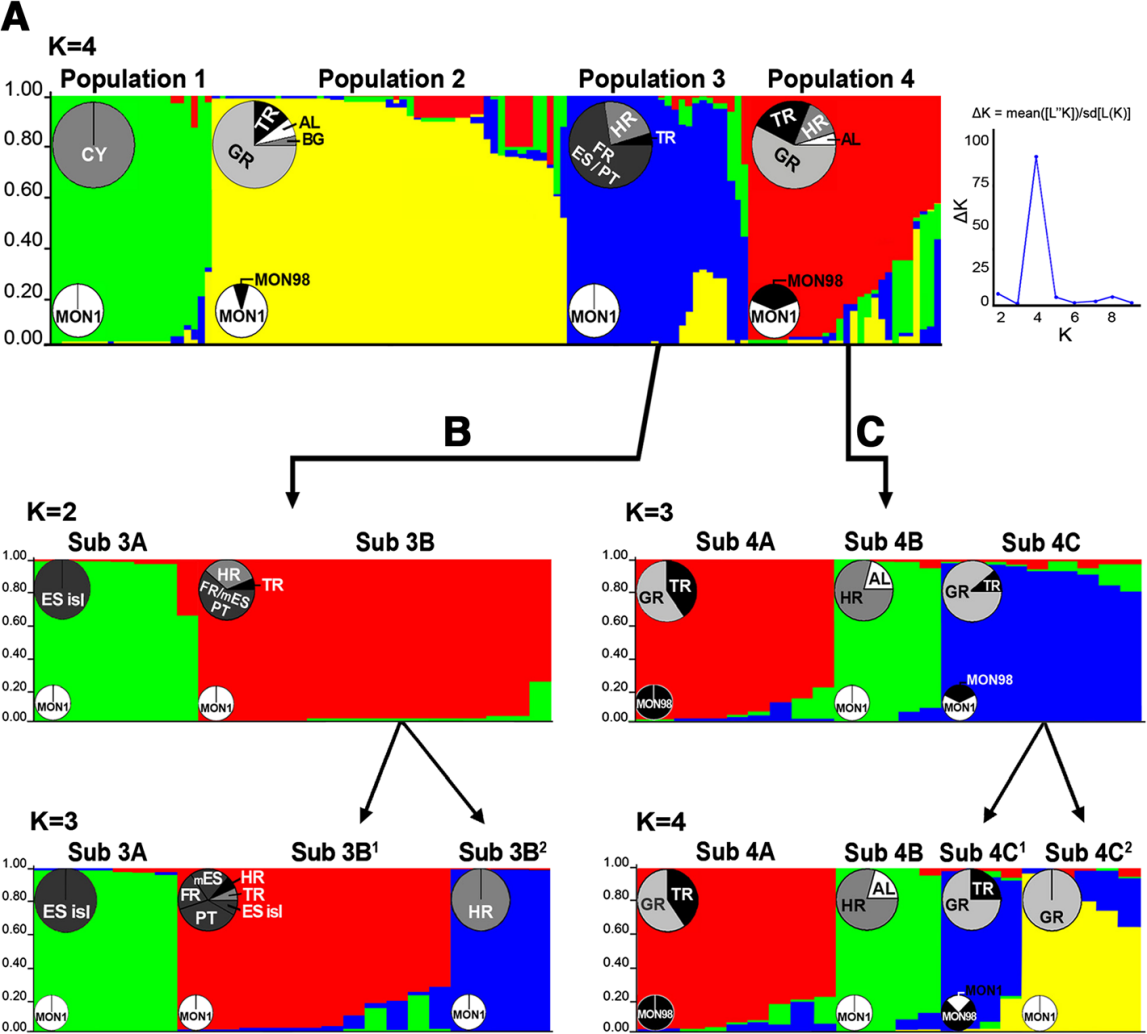
**Population structure of the 128** ***L. infantum *strains inferred by STRUCTURE analysis.** Population structure is based on the MLMT data observed for each analysed strain at 14 microsatellite markers. Each population is represented by different colour and differently coloured segments in the vertical bar correspond to the membership of each strain to different populations. The length of each coloured segment is proportional to the estimated membership coefficient of each strain per population. The percentage of strains enclosed per population/subpopulation based on their geographical origin (TR, Turkey; CY, Cyprus; BG, Bulgaria; GR, Greece; AL, Albania; HR, Croatia; FR, France; ES, Spain; PT, Portugal) and *L. infantum* zymodeme (MON-1, MON-98), is demonstrated by large and small pie-charts, respectively. **A.** The likelihood of population number was calculated by STRUCTURE HARVESTER [64] implementing the Evanno method [63]. The major peak at K = 4 at the ΔK graph indicates the existence of four populations (populations 1–4) for the total strain set, which were constantly observed in all independent simulations (10 runs). Population subdivision was investigated by re-analyzing separately each population at K = 4; subdivision of populations 1 and 2 is not shown. **B.** Three geographically determined subpopulations (subs 3A, 3B^1^ and 3B^2^) were observed upon re-analysis of population 3. Bar plots for both K = 2 and K = 3 are shown. **C.** Four stable subpopulations (subs 4A, 4B, 4C^1^ and 4C^2^) based on both geographical origin and zymodeme type, were identified for population 4. Bar plots for both K = 3 and K = 4 are shown.

Calculation of ΔK indicated the existence of four distinct genetic populations (Figure 2Α ). **Population 1** (23 strains, all MON-1) comprised all strains (canine isolates) from Cyprus (CY). **Population 2** (51 strains) was formed by half of the strains from Turkey (8 TR), the single isolate from Bulgaria (BG) and the majority of strains from Greece (GR) and Albania (AL) (38 GR and 4 AL). Five of these strains belonged to zymodeme MON-98 and were isolated from dogs in remote places of Greece, while all other were MON-1 strains. **Population 3** (26 strains, all MON-1) incorporated all strains from SW Europe (19 FR/ES/PT), more than half of the strains from Croatia (6 HR) as well as the only TR strain isolated in the Black Sea region. **Population 4** (28 strains, MON-1 and MON-98) enclosed the remaining TR (7), GR (16), AL (1) and HR (4) strains. Sixteen out of the 21 MON-98 strains from Kuşadasi (Turkey) and Crete (Greece) were assigned to this population. Population assignment per analyzed strain at K = 4 is designated also in Figure 1 and Table 1.

**Figure 3:**
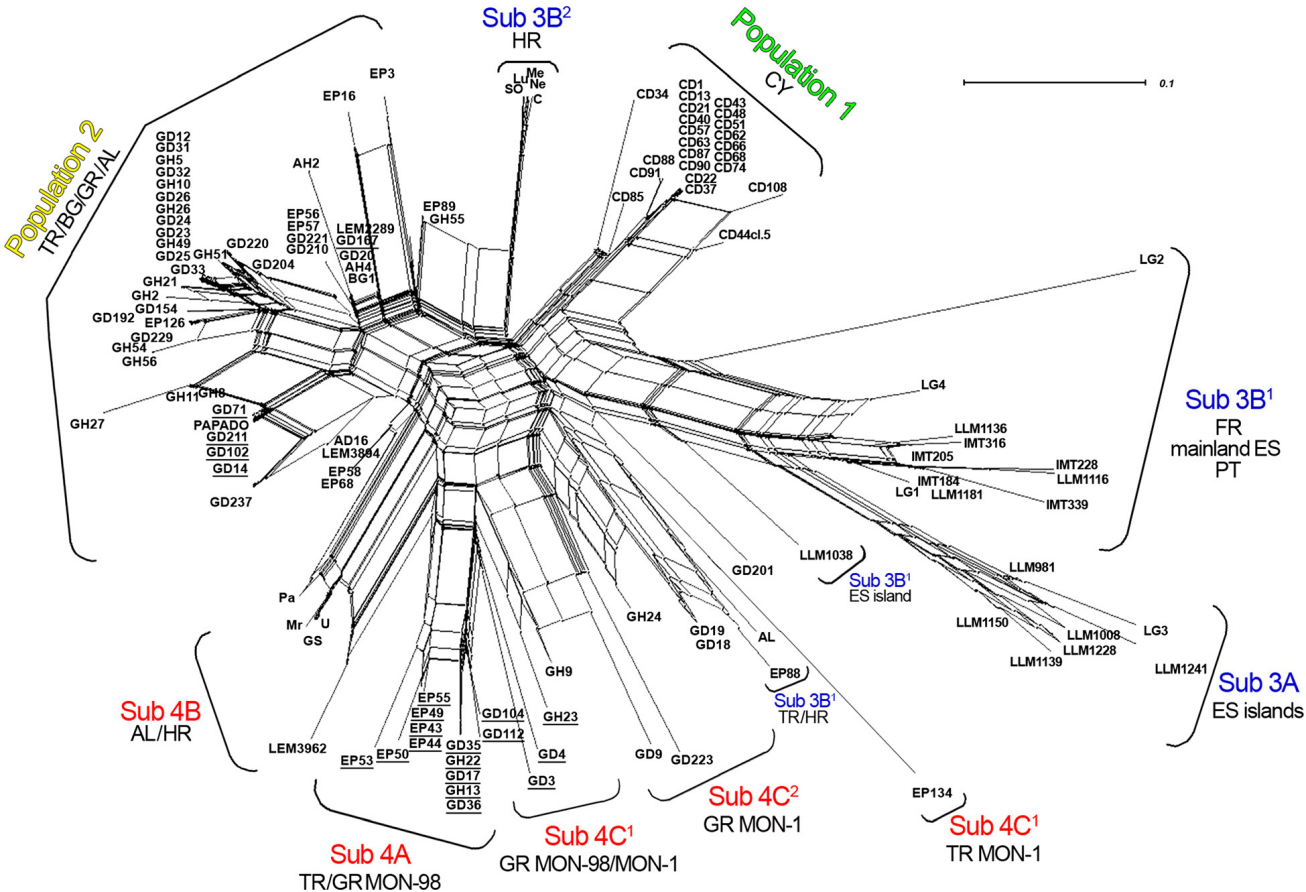
**Phylogenetic network (NeighborNet) constructed by Splitstree for the 128** ***L. infantum *strains studied.** The NeighborNet algorithm detected the same population substructures inferred by STRUCTURE (Figure 2, Table 1) for population 3 (Sub3A, Sub3B^1^ and Sub3B^2^) and population 4 (Sub4A, Sub4B, Sub4C^1^ and Sub4C^2^). Subdivision of populations 1 and 2 is not supported by NeighborNet analysis. TR, Turkey; CY, Cyprus; BG, Bulgaria; GR, Greece; AL, Albania; HR, Croatia; FR, France; ES, Spain; PT, Portugal; MON-1, *L. infantum* zymodeme MON-1; MON-98, *L. infantum* zymodeme MON-98. Strains that presented identical MLM Types (Table 1) are underlined. Strains are represented by coloured boxes that correspond to their population or subpopulation assignment according to STRUCTURE analysis (Figure 2).

**Figure 4:**
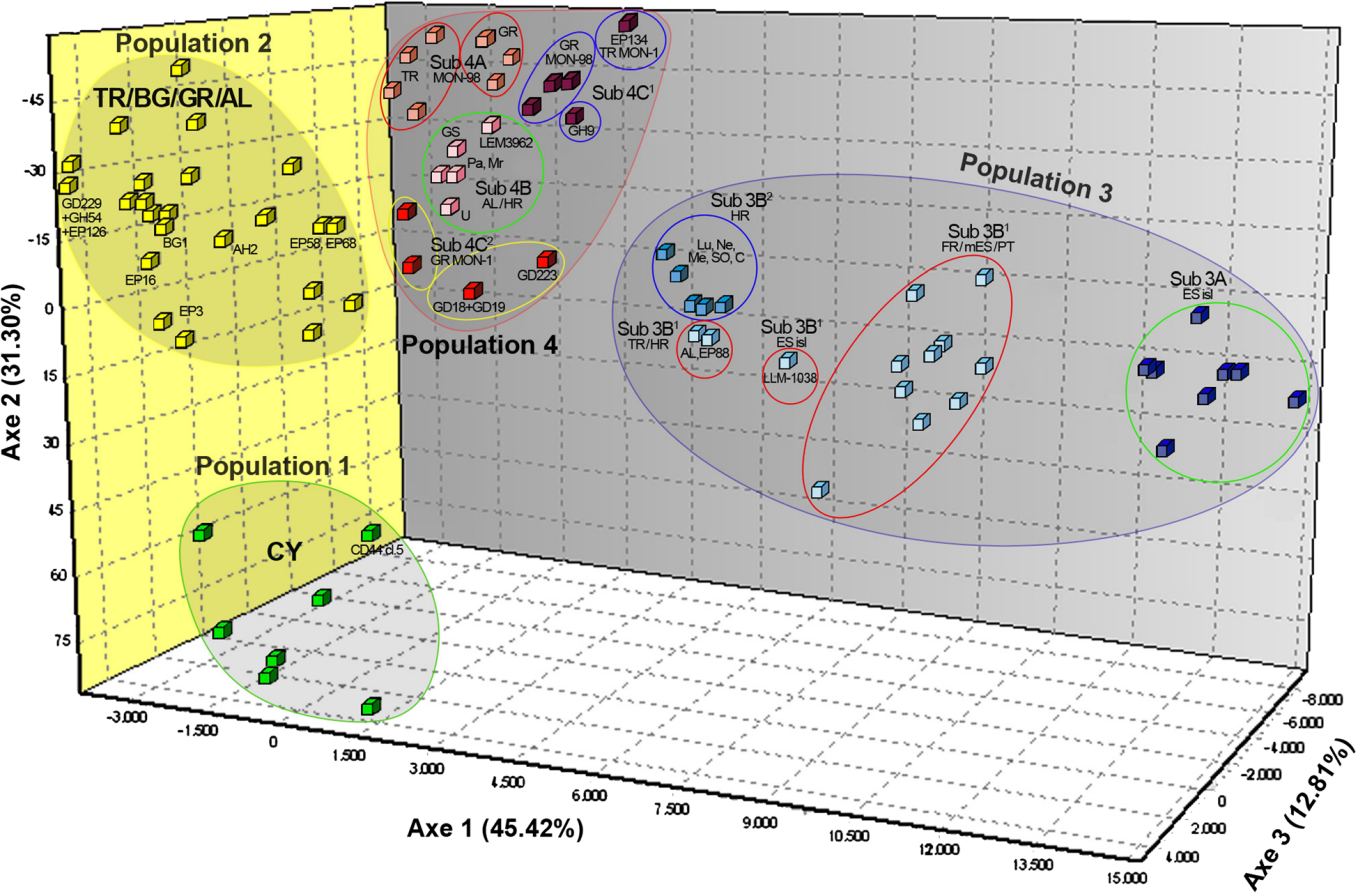
**Spacial distribution of the 128** ***L. infantum *strains by factorial correspondence analysis (FCA).** Subdivision of population 1 (green circle) and population 2 (yellow circle) is also not demonstrated in the FCA plot. The subpopulations observed at STRUCTURE (Figure 2B and 2C) and NeighborNet analysis (Figure 3) for population 3 (blue circle) and population 4 (red circle) correspond to those illustrated by FCAnalysis. These are Sub3A (dark blue squares), Sub3B^1^ (light blue squares), Sub3B^2^ (blue squares), Sub4A (dark pink squares), Sub4B (light pink squares), Sub4C^1^ (dark red squares) and Sub4C^2^ (red squares). TR, Turkey; CY, Cyprus; BG, Bulgaria; GR, Greece; AL, Albania; HR, Croatia; FR, France; ES, Spain; PT, Portugal; MON-1, *L. infantum* zymodeme MON-1; MON-98, *L. infantum* zymodeme MON-98. Strains sharing the same MLM Type (Table 1) are not shown. The colours of the circles designating each subpopulation correspond to those in Figures 2B, 2C and Figure 3.

Population structure at K = 4 was well-established in all ten iterations and different genetic distance analyses (Figures 3, 4 and Additional file 1: Figure S1) corroborated the existence of the four populations determined by the model-based analysis. However, the assignment of HR strains to **POP3** and **POP4** by STRUCTURE analysis at K = 4 (Figure 2Α ) was found inconsistent with their placement at the mid-point rooted NJ tree (Additional file 1: Figure S1) as well as with the distribution of the respective splits in the phylogenetic network (NeighborNet) (Figure 3). The FCA plot also confirmed the existence of at least four main populations (Figure 4). In overall, the three-dimensional representation mirrored the genetic isolation of CY strains (**POP1**), the differentiation of SW European strains (**POP3**) from all other populations as well as between the Balearic Islands and FR/mainland ES/PT, and the diversification of TR/HR strains from SW European strains.

The population structure defined by STRUCTURE was however, unstable for other K values and subdivision was detected by re-analyzing these four populations independently. **Population 1** split further in two subpopulations (not shown), but subdivision was not supported by other genetic distance analyses (Figures 3, 4 and Additional file 1: Figure S1).

**Population 2** was divided in three subpopulations (not shown); however there was no correlation between subpopulations and host specificity, as both human and canine isolates were found in every subcluster. Additionally, the assignment of strains to these three subpopulations was not related to their geographical origin as strains from both mainland and coastal areas of TR and GR never divided, and the GR strains always grouped with those from BG and AL. Moreover, no differentiation based on zymodeme type was detected between GR MON-98 strains and BG/GR/AL MON-1 strains. In addition, the subdivision of **POP2** was not supported by the different distance-based analyses used (Figures 3, 4 and Additional file 1: Figure S1).

**Population 3** was divided in three stable subpopulations (Figure 2B). All but one strain from the islands of ES were assigned to a distinct subpopulation (**sub3A**), as previously observed [15]. **Sub3B**^**1**^ enclosed all strains from FR, PT and the mainland of ES as well as single strains from TR, HR and the ES island of Majorca. Of note is that the three strains from TR, HR and Majorca that grouped with strains from the mainland ES in **sub3B**^**1**^ (Figure 2B), were placed in intermediate positions at the Splitstree and FCA plots (Figures 3 and 4) and that the single strains EP88 and AL from TR and HR respectively, formed a separate subgroup distant from other strains of **POP3** at the NJ tree (Additional file 1: Figure S1). Moreover, strains EP88 and AL presented large splits in the phylogenetic network (Figure 3) and were found more closely related to a group of TR/GR isolates (strains EP134, GD18 and GD19) that were assigned at **POP4** by STRUCTURE. This finding is also illustrated in the FCA plot (Figure 4). Accordingly in the NJ tree, strains EP88 and AL branched out and clustered with the same group of TR/GR strains after the formation of **POP1** (Additional file 1: Figure S1). All other strains from HR (Lu, Ne, Me, SO and C) grouped in a separate homogeneous subpopulation (**sub3B**^**2**^) (Figure 2B), which was supported by all analysis methods (Figure 3, 4 and Additional file 1: Figure S1).

Re-analysis of **population 4**, which included the majority of the MON-98 strains, revealed further sub-structuring (4 stable subpopulations) based on both zymodeme type and geographical origin (Figure 2C). **Sub4A** included only MON-98 strains from Turkey and Greece, while the Splitstree plot (Figure 3) illustrated gene flow between them. The group of HR strains (Pa, Mr, GS and U) and one strain from Albania (LEM3962), which was assigned to **sub4B,** presented a reticulate pattern in the Splitstree network (Figure 3) rather closely related to two TR strains (EP58 and EP68) which were assigned to **POP2**. This was also apparent in the NJ tree (Additional file 1: Figure S1) and the FCA plot (Figure 4). Turkish and Greek MON-1 and MON-98 strains were found in **sub4C**^**1**^, whereas **sub4C**^**2**^ enclosed only GR MON-1 strains, most of which presented the 626 bp *K26* amplicon (Table 1).

### Genotypic characterization and F-statistics for populations and subpopulations

The measures of genetic diversity calculated for each main population defined by STRUCTURE are summarized in Table 4. Out of the 128 analyzed strains, 50 strains presented unique microsatellite profiles and 78 strains shared the same MLM Type with at least another strain. Identical profiles were found only among strains that were assigned in the same main population (Tables 1 and 4). The genetic polymorphism exhibited per analyzed locus for each population is given in Additional file 2: Table S1.

**Table 4:** **Descriptive statistics by population (POP) and comparison of the F**_***IS ***_**values between the four main populations defined by STRUCTURE analysis**

**POP**	**Origin**	**Total no of MLMT profiles**	**P**	**A**	**H**_***e***_	**H**_***o***_	**F**_***IS***_
**1** (23)	CY	6	0.36	1.36	0.03	0.01	0.72
**2** (51)	TR, BG, GR, AL	23	0.64	2.29	0.12	0.02	0.87
**3** (26)	TR, HR, FR, ES, PT	22	0.86	2.71	0.28	0.02	0.91
**4** (28)	TR, GR, AL, HR	20	0.71	2.86	0.24	0.04	0.82
**Mean**			**0.64**	**2.30**	**0.17**	**0.02**	**0.86**

The data are based on the analysis 128 *L. infantum* strains by 14 microsatellite markers. The number of strains assigned per population is shown in parenthesis. Origin acronyms indicate the strain geographical source. P, proportion of polymorphic loci; A, number of alleles; H_*e*_, Nei’s unbiased expected heterozygosity; H_*o*_, observed heterozygosity; F_*IS*_, inbreeding coefficient.

All strain populations were polymorphic at markers Lm2TG, Lm4TA, Li22-35 (E), Li23-41 (F) and CS20. The highest degree of polymorphism and allelic richness in all markers was observed within **POP3** and **POP4**, where markers LIST7039 and Li71-33 (P) were also polymorphic. Comparable variation was detected within **POP2**, although this population included twice the number of strains comprising **POP3** and **POP4**. In contrast, **POP1**, exclusively formed by CY strains, presented the lowest polymorphism and allelic variation. Strains within **POP2** and **POP3** further segregated at markers LIST7031 and Li71-5/2 (Q). Similarly, marker Li41-56 (B) was polymorphic only among strains in **POP2** and **POP4**. All main populations were characterized by much lower observed heterozygosities than the expected ones (mean H_*e*_ = 0.17 and mean H_*o*_ = 0.02) as well as high inbreeding coefficients (mean F_***IS***_ = 0.86) (Table 4).

Regarding the subpopulations defined by STRUCTURE and largely supported by the genetic distance analyses, the lowest levels of polymorphism, heterozygosity and inbreeding were found in **sub3B**^**2**^ (HR strains) and **sub4B** (AL/HR strains) (Additional file 3: Table S2). The fact that lower F_***IS***_ values are observed at subpopulation level indicate that Wahlund effect (population subdivision) accounts at least partially to the high inbreeding coefficients found for the main populations.

The four populations identified by model- and distance-based analyses were further supported by *F*-statistics (Table 5) indicating strong differentiation between them. CY strains that grouped in **POP1**, demonstrated the greatest genetic isolation from all other populations (F_*ST=*_0.546-0.655). As shown in Additional file 4: Table S3, the observed subpopulations were also strongly supported by F-statistics (*p* ≤ 0.006).

**Table 5:** **F**_**ST **_**values and corresponding *p*-values for the populations identified at K = 4**

**Populations**	**1**	**2**	**3**	**4**
**1** (23)	0	0.655	0.546	0.558
**2** (51)	<0.001	0	0.526	0.409
**3** (26)	<0.001	<0.001	0	0.358
**4** (28)	<0.001	<0.001	<0.001	0

Population assignment is based on STRUCTURE analysis; In parenthesis, the number of strains comprising each population; F_ST_, pairwise Wright’s fixation index (upper right triangle); *p*-values, confidence test (lower left triangle).

### Correlation of MLMT profiles with origin, zymodeme type and host

The strain-set was also grouped based on geographical source and their MLM Types were analyzed to compare the degree of polymorphism between and within each endemic country. At least one matching pair was detected within each endemic country (Table 1).

Notably, the same MLMT profile (**No 29**) was identified for 17 out of 23 canine isolates from different prefectures of Cyprus and a second one (**No 33**) was shared by two canines from Paphos prefecture. Seven distinct profiles (**No 45–51**) were identified for the ten analyzed strains from Croatia. Of these, three multilocus genotypes (**No 45, 47** and **48**) were observed in more than one Croatian strain. Two canine isolates from Albania were indistinguishable by MLMT (**No 54**). The same MLMT profile (**No 37**) was identified among MON-98 canine isolates from Kuşadasi district (Aegean region), whereas a second one (**No 41**) was shared by strains of unknown zymodeme (EP58 and EP68) that were isolated from dogs in coastal areas of the Aegean (Muğla and Kuşadasi, respectively).

Only 20 out of 54 strains analyzed from Greece presented distinct MLM Types (Table 1). The same MLMT profile (**No 2**) was shared by MON-1 strains (human and canine isolates) from the capital of Athens, the island of Mytilene and Lasithi prefecture in Crete. An identical profile (**No 22**) was shared by MON-1 strains (canine isolates) from Rhodes island and the capital of North Greece Thessaloniki, where the second largest seaport is located. Another two MLMT profiles **(No 5** and **19**) were identical between either MON-98 strains or MON-1 strains from Crete. MLM Type **No 3** was common between MON-1 strains (human isolates) from Athens and MON-98 strains (canine isolates) from a harbor area in Attica, northern Peloponnese and Crete.

Furthermore, identical MLM Types were identified between strains originating from different countries, except for strains from Croatia and Cyprus. Notably, the single canine isolate from Bulgaria shared the same MLMT profile (**No 11**) with one Albanian and three Greek strains. Also, two MLM Types (**No 13** and **No 25**) were common between strains from Greece and Turkey. One of them was found in canine isolates from the Aegean region (Cyclades-Greece and Bodrum-Turkey, respectively) and a human isolate from Central Greece. The other MLM Type was shared solely by MON-1 canine isolates from north inland regions of Greece and Turkey and one strain of unknown zymodeme from Muğla (Aegean region). As shown in the Splitstree plot, these strains were separated in a clonal subgroup (Figure 3).

Characteristic alleles only for CY isolates were identified at markers Lm2TG and Lm4TA. Also, HR strains presented distinct alleles at marker Li71-7 (R) and GR strains at markers Li22-35 (E) and Li23-41 (F). No correlation was observed between specific microsatellite profiles and human or canine hosts.

## Discussion

The aim of the present study was to evaluate epidemiological aspects of *L. infantum* causing VL in the SE edge of Europe focusing on Turkey, Cyprus and Greece but also including strains from Balkan countries.

Initially, the *K26*-PCR assay was applied to confirm the presence of *L. infantum* spp. and simultaneously to distinguish MON-1 from non MON-1 zymodemes within the strain-set [14]. Application of the *K26* typing tool, which discriminates between different *L. infantum* and *L. donovani* zymodemes, was also essential due to the recent detection of *L. donovani* MON-37 in Cyprus [35] as well as the identification of the novel *L. donovani* MON-308 and MON-309 zymodemes in Turkey [36]. Notably, strains belonging to these three *L. donovani* zymodemes were found by MLMT to form a novel *L. donovani sensu lato* group [36].

In consistence with previous findings [14], *K26* typing revealed that all strains from SE Mediterranean and Balkan countries were *L. infantum* presenting single *K26* amplicons of 626 bp or 940 bp, except for two Greek MON-1 strains showing both *K26-*PCR products. In line with *K26* typing, MLEE analysis confirmed the predominance of zymodeme MON-1. Of the 70 strains isolated from the Aegean region of Turkey, mainland regions of Greece and the island of Crete, 21 (30%) were typed, however, as zymodeme MON-98. This is the highest percentage of MON-98 strains found so far [5,14,69] and it should be noticed that Greek MON-1 and MON-98 strains were similarly distributed in the country. Since these are the only zymodemes found so far in the Balkans, the K26-PCR assay is adequate for *Leishmania* typing in this endemic region.

The MLMT profiles obtained for 109 *L. infantum* MON-1 and MON-98 strains from six SE European countries were compared to those of 19 previously analyzed *L. infantum* MON-1 strains from SW Europe (France, Spain and Portugal) [15,52,61]. A small number of Greek MON-1 and MON-98 strains and two Turkish *L. infantum* strains of unknown zymodeme type (strains EP3 and EP16) (Table 1) had been also analyzed earlier [15,36,52,55,61]. In this study we have investigated additional strains from VL foci in Turkey and Greece and also included *L. infantum* strains from Cyprus, Bulgaria, Albania and Croatia, for which genetic diversity and population structure had not been studied until now. By this we could confirm the existence of substantial differentiation between *L. infantum* strains from the eastern and western parts of south Europe, and that population structuring is geographically determined, as previously demonstrated [70]. These findings are also in agreement with previous data according to which, Cypriot and Iberian strains could be distinguished upon processing their *K26*-PCR products by RFLP [14].

Different types of population genetic analyses, including Bayesian inference (as implemented in STRUCTURE), distance-based (NJ and Neighbor Net in SplitsTree) and FCA revealed the existence of four putative populations. Population 1 was well-defined by all methods and consisted exclusively of the Cypriot strains of *L. infantum* and was clearly separated from the other populations (Figures 3 and 4). As expected, all SW European strains formed a distinct population (POP3). Bayesian statistics has also assigned one strain from the Black Sea area in Turkey and six strains from Croatia to this population that was, however, not well-supported by the distance-based and FCA analyses, which indicates a rather intermediate position of these strains. Three subpopulations became apparent by separate re-analysis. The differentiation between strains from the Balearic Islands (sub3A) and strains from the Iberian Peninsula and France (sub3B^1^) confirms previous analysis [15,52,61]. All but one strain from Croatia that grouped in POP3, were assigned to the third subpopulation (sub3B^2^). The Turkish and Greek strains split among two populations, POP2 and POP4 with no correlation to their place and year of isolation, the zymodeme type, host specificity or pathology. POP2 was composed by most strains from Turkey, Greece, Albania and the single strain from Bulgaria. Nevertheless, distance and FCA analyses revealed that this population was rather homogenous and was not divided into subpopulations. In contrast, POP4 that enclosed some Turkish/Greek, one Albanian and four Croatian strains, appeared to be highly diverse and consisting of four subpopulations. Sub4A was formed only by Turkish/Greek MON-98 strains and sub4B enclosed only Albanian/Croatian strains. The other Turkish/Greek strains, whether of zymodeme MON-1 or MON-98, were assigned to sub4C^1^ and sub4C^2^.

In general, model- and distance- based algorithms identified the same population and subpopulation divisions. The statistical support for the midpoint-rooted NJ tree was assessed by bootstrap analysis. However, sufficient bootstrap values were only observed for nodes between groups of strains found within the main clusters or sub-clusters (Additional file 1: Figure S1), while a spider-net pattern and large splits were often observed in Splitstree (Figure 3), probably due to the existence of homoplasy, intermediate or mixed genotypes and considerable genetic migration. This was expected, since the analyzed strains belong to genetically close *L. infantum* zymodemes and were isolated from geographically close countries with frequent movements of host populations and could explain the discrepancies found in the assignment of some groups of strains or individual strains by different model- and distance-based approaches. Analogous congruity has been also observed elsewhere [15,52].

Compared to previous studies [15,36,52,55,61], we have now analyzed a much larger number of MON-1 and MON-98 strains from Turkey and Greece, but as before, we were unable to differentiate these strains according to their geographical origin, Turkey vs. Greece or Greek mainland vs. the island of Crete, or between MON-1 and MON-98 strains. Most MON-98 strains from both Turkey and Greece were assigned to the highly diverse POP4 together with strains of zymodeme MON-1 from the same areas, but clustered in the two subpopulations sub4A and sub4C^1^ (Figure 2C). A distinct subpopulation of the MON-98 strains that grouped in POP2 was not supported by distance-based analyses. The reticulate network in Splitstree (Figure 3) clearly points to extensive gene flow and/or hybridization occurring among Turkish and Greek strains of both zymodemes. Our results were not affected when a group of representative non MON-1 strains, other than those of zymodeme MON-98, was included in our analysis (not shown). This finding is in agreement with previous analysis [15,36,52,55,61], according to which a small group of analyzed MON-98 strains from Crete did not cluster with other non MON-1 strains from SW Europe but always remained part of the MON-1 population. Our observations reflect the high polymorphism observed among different groups of *L. infantum* MON-1 strains rather than indicate differentiation between MON-1 and MON-98 zymodemes.

Identical MLM Types were found between MON-1 strains from Turkey and Greece, which along with the observed shared membership of POP2 and POP4 and the significantly high inbreeding level within TR/GR MON-1 (F_*IS*_ ≥ 0.873) indicate considerable recombination and gene flow among them as previously suggested [15]. These findings may be associated with intensive host migration over long periods of time and common eco-epidemiological features, including a similar sand fly fauna [71], in Turkey and Greece. The detection of identical MLMT genotypes between Greek strains of both zymodemes in harbor areas of the mainland and in Greek islands suggests parasite transfer between the mainland and the islands.

The assignment of Greek human and canine isolates to the same genetic populations indicates the absence of host specificity. Since we have included a much larger number of strains, this observation enhances previous findings [15].

The genetic structure of strains from Crete appears to be a micrograph of the overall epidemiology of VL in Greece, which is in contrast to the clear division between strains from the mainland and islands of Spain, Majorca [8] and Ibiza [15]. At high hierarchy, the overall high genetic diversity, significant gene flow and population subdivision have most probably resulted from successive recombination or even hybridization events. In parallel, the observed low variability measures and considerable inbreeding observed within small subgroups of MON-1 or MON-98 strains from either inland regions of Greece or the island of Crete (Table 3) can be explained by successive subdivision followed by genetic drift within each of these groups and could indicate a founder effect. The existence of related clones of MON-1 as well as MON-98 strains could indicate selection of specific microsatellite genotypes, as previously observed for MON-1 strains from Madrid or Majorca and Ibiza [72].

*L. infantum* MON-1 canine strains from Cyprus were found genetically isolated from all other genetic groups and formed a monophyletic group (Additional file 1: Figure S1) presenting the lowest degree of polymorphism, allelic deprivation and traces of heterozygosity, which indicate genetic drift. Seventeen out of the 23 strains shared the same MLMT genotype suggesting an epidemic outbreak due to the same strain which has spread to different parts of the island. As expected the clone of strain CD44 (CD44cl.5) shared common alleles and grouped with the other MON-1 strains from Cyprus. The identification of different allelic variants than those typically found for *L. donovani* MON-37 strains [35], responsible for both HVL and CL, suggests the absence of recombination or hybridization between MON-1 and MON-37 strains. The epidemiology of CanL in Cyprus has been doubtless affected by the eradication campaigns held against malaria between 1940 and 1950 which have massively reduced the vector populations on the island. Another campaign was performed against echinococcosis between 1970 and 1975 which diminished the dog populations and altogether eliminated CanL for over 20 years [11]. Most probably this has caused severe bottleneck events in *Leishmania* populations leading to a limited parasite genetic pool characterized by significant inbreeding and genetic homogeneity. However, after the sand fly fauna has gradually recovered and CanL re-emerged, some degree of genetic variation was observed in the parasites as a result of adaptation to selective pressure.

The partition of canine MON-1 strains from Croatia between both SE (POP4) and SW (POP3) European populations (Figures 2, 4 and Additional file 1: Figure S1) mirrors the geographical position of the country. Despite the relatively small number of strains, we could document substantial genetic diversity for these strains. Croatia is situated between East and West Europe and parasites can be introduced from its neighboring countries. The microclimate, the presence of vectors and reservoirs favor the establishment and circulation of parasites in the Dalmatian coastal regions. The exportation from Croatia to neighboring countries is equally possible, as adult VL cases acquired at the Dalmatian coast were registered in Austria and Switzerland [40,42]. The genetic differentiation observed between Croatian and SW European strains on one side and between Turkish, Greek and Croatian strains, on the other side, can be also seen by the distinct and intermediate placement of the Croatian strains in the Splitstree (Figure 3). The rather low level of inbreeding and the substantially low variability values observed within these subpopulations (Tables 4 and Additional file 4: Table S3) indicate genetic drift, most probably caused by population substructure (Wahlund effect). Other explanations could be the restriction of the parasites’ gene pool due to mass insecticide spraying for malaria control or the diversification of autochthonous cases circulating in Croatia for a long time [42]. Considering the unexpected genetic diversity of Croatian strains isolated from a rather small territory, future investigations should use a larger sample set including human and sand fly isolates, and should include strains from the northern Adriatic region and Italy.

Strains from Albania did not form a distinct genetic group in our analyses, but grouped either with strains from Turkey/Greece or Croatia. In contrast, a considerable amount of genetic flow was detected obviously reflecting the frequent human migration and the unrestrained movement of canine and sand fly populations between these countries. HVL in Albania still remains an infantile disease and presents by far the highest morbidity rates in Europe [45] although relapses and resistance to drug treatment are only rarely reported [44-46]. It raises concerns that 57.1% of seropositive HVL cases hospitalized in the Ioannina prefecture (North Greece) are immigrants from the neighboring villages in south Albania and that the highest prevalence of clinically affected HVL (42.8%) and CanL (82.8%) in Greece is reported in the Thesprotia prefecture (Epirus) close to the border between Greece and Albania [31]. These findings are alarming for both countries and suggest that the dynamics and complexity of VL in Albania could be similar to those observed in Greece. CanL is endemic across the country with an estimated 16-17% seroprevalence, while sand fly species *Phlebotomus neglectus, Phlebotomus perfiliewi* and *Phlebotomus tobbi* are widespread. It should not be underestimated that the maximum incidence of *P. neglectus*, which is highly present in all Greek islands except Gavdos [5,71] and is a proven *L. infantum* vector in the island of Corfu neighboring the Albanian coast [44], is reported in endemic foci presenting the highest VL morbidity rates. It should also be noticed that *P. tobbi* spp. despite its overall low abundance, is solely found in Albanian districts where VL morbidity exceeds 1/1000 [44].

The lack of isolated *Leishmania* strains from Bulgaria has so far restricted the investigation of the population structure and dynamics. We have included in our study the first strain that was isolated from a dog in the Blagoevgrad province (southwestern Bulgaria) that borders the Macedonia region of Greece (Figure 1). Our findings confirm the existence of at least *L. infantum* MON-1 zymodeme in Bulgaria [48] and demonstrate that the MLM Type of this particular canine isolate is identical to that of three strains from Greece and one strain from Albania. This is not surprising as Bulgaria shares common geo-ecological characteristics with its neighboring countries. The presence of *L. infantum* sand fly vectors and stable parasite circulation in Stara Zagora [49] highlights the necessity to conduct a comprehensive entomological and epizootic survey for the identification of *Leishmania* vectors and the isolation of *Leishmania* parasites from sand flies, human and canine hosts. This would give the opportunity to thoroughly investigate the population structure of *L. infantum* spp. and disease dynamics in Bulgaria.

## Conclusions

This is the first study that gives an insight into the population structure of *L. infantum* in Cyprus located at the SE edge of Europe and the Balkans (Turkey, Bulgaria, Greece, Albania and Croatia). *L. infantum* MON-1 was found to be predominant in this region, while 16.8% of strains were typed as MON-98. Hence, the *K26* typing tool proves adequate for typing strains from SE Europe and the Balkans. Herewith, we have identified distinct genetic populations that reveal the clear differentiation between SE and SW European strains, the intermediate placement and further diversification of Croatian strains from SW strains and the formation of a monophyletic group enclosing all canine MON-1 isolates from Cyprus. The latter could be explained by the natural isolation of Cyprus being an island and a bottleneck effect caused by the subsequent eradication campaigns against malaria and echinococcosis that were held in Cyprus. Also, we have analysed the highest number of MON-98 strains found so far in Turkey and Greece and did not identify a distinct population including all MON-98 strains. The latter finding is strengthened by the separate analysis of MON-98 and MON-1 strains, which shows that the genetic distances observed between MON-98 and MON-1 subgroups are similar to those found between different MON-1 subgroups. Altogether our results confirm and extend the study by Kuhls *et al*. [15] using a larger strain-set from Turkey and Greece and including strains from other endemic Balkan countries. Notably, the clear diversification between Spanish strains from the mainland and the islands of Majorca and Ibiza is contrary to the clustering of Greek strains from the mainland and the islands of Corfu, Crete, Mytilene and Rhodes. Overall, the observed structuring is associated with the sand fly fauna, geo-ecological characteristics, historical background and important events that have influenced the epidemiology of visceral leishmaniasis in these endemic countries.

## Competing interests

The authors declare that they have no competing interests.

## Authors’ contributions

EG, CH, GS and KS conceived and designed the study. EG carried out the experiments and analysed the data. EG, CH, DS, GS and KS drafted and revised the manuscript. MA, VC, FM, TŽ, FP, J-P D, YÖ, SÖT, WP provided *Leishmania* strains, analysis tools and/or contributed to the critical revision of the manuscript. All authors read and approved the final version of the manuscript.

## Supplementary Material

Additional file 1: Figure S1 Midpoint rooted Neighbor-joining tree constructed for the 128 *L. infantum* strains studied. The midpoint rooted tree is based on the Dps-distances calculated for the MLMT data at 14 microsatellite markers for the total strain set. Bootstrap values only above 50% are indicated at key nodes. *L. infantum* zymodemes, geographical origins and populations as inferred by STRUCTURE at K=4 (Figure 2A) are shown in colored boxes next to the tree. The colors designating each population correspond to those in Figures 2A, 3 and 4. TR, Turkey; CY, Cyprus; BG, Bulgaria; GR, Greece; AL, Albania; HR, Croatia; FR, France; ES, Spain; PT, Portugal. Click here for file

Additional file 2: Table S1 Descriptive statistics by microsatellite locus for each of the main four populations defined by STRUCTURE analysis. Click here for file

Additional file 3: Table S2 Descriptive statistics and comparison of F_*IS*_ values between the sub-populations defined by STRUCTURE. Click here for file

Additional file 4: Table S3 F_ST_ values and corresponding *p*-values for the sub-populations identified by STRUCTURE re-analysis. Click here for file
