# Supplementary figures and images for: Genetic diversity and structure in Leishmania infantum populations from southeastern Europe revealed by microsatellite analysis
Source: Parasit Vectors. 2013 Dec 5;6:342. doi: 10.1186/1756-3305-6-342 (PMC4029556; doi:10.1186/1756-3305-6-342)

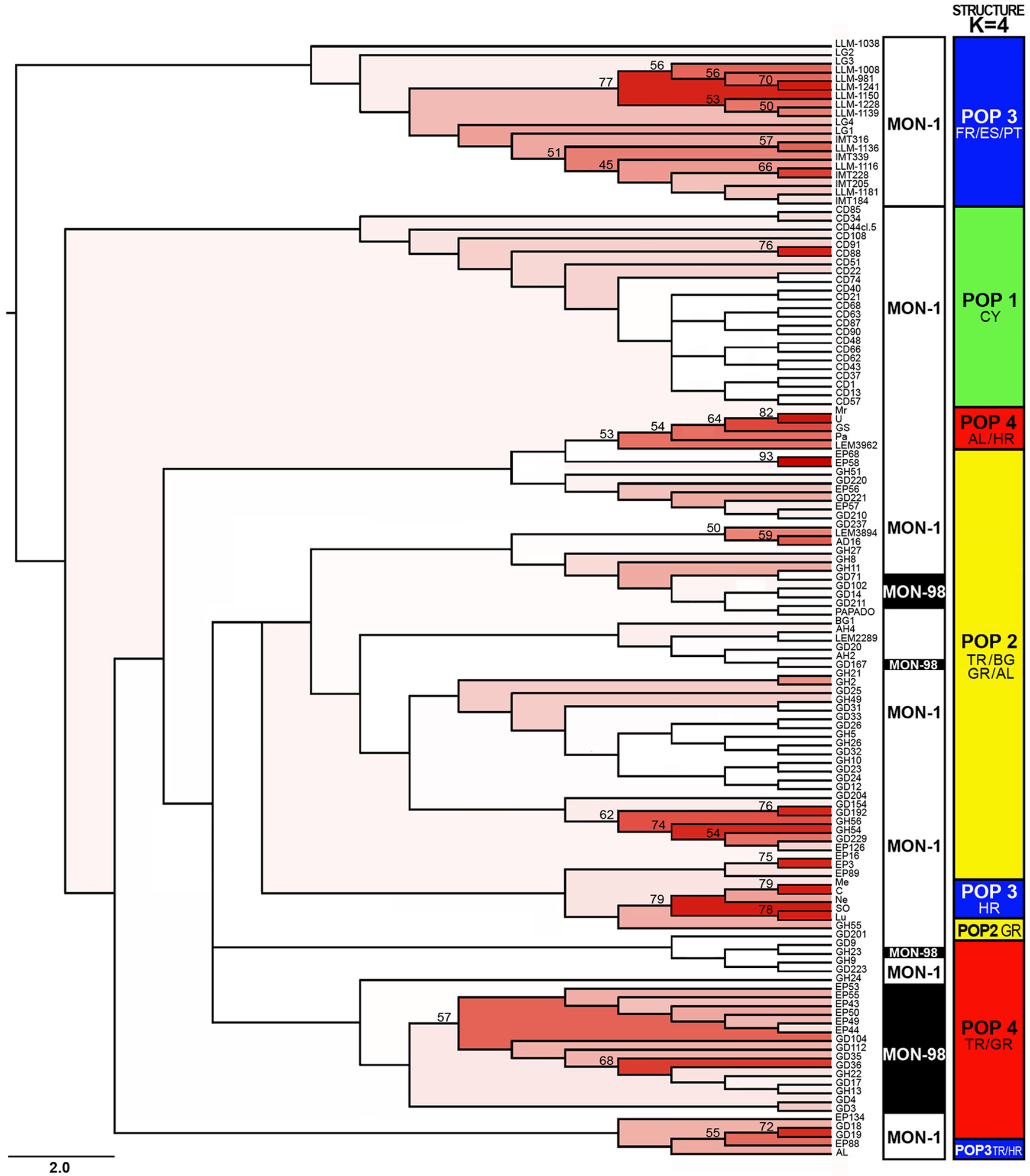

Supplement: Additional file 1: Figure S1 — Midpoint rooted Neighbor-joining tree constructed for the 128 L. infantum strains studied. The midpoint rooted tree is based on the Dps-distances calculated for the MLMT data at 14 microsatellite markers for the total strain set. Bootstrap values only above 50% are indicated at key nodes. L. infantum zymodemes, geographical origins and populations as inferred by STRUCTURE at K=4 (Figure 2A) are shown in colored boxes next to the tree. The colors designating each population correspond to those in Figures 2A, 3 and 4. TR, Turkey; CY, Cyprus; BG, Bulgaria; GR, Greece; AL, Albania; HR, Croatia; FR, France; ES, Spain; PT, Portugal. [file 1756-3305-6-342-S1.jpeg]
