# Supplementary material for: Genetic diversity and structure in Leishmania infantum populations from southeastern Europe revealed by microsatellite analysis
Source: Parasit Vectors. 2013 Dec 5;6:342. doi: 10.1186/1756-3305-6-342 (PMC4029556; doi:10.1186/1756-3305-6-342)
Supplement: Additional file 2: Table S1 — Descriptive statistics by microsatellite locus for each of the main four populations defined by STRUCTURE analysis. [file 1756-3305-6-342-S2.doc]

**Additional File 2**

**Table S1. Descriptive statistics by microsatellite locus for each of the main four populations defined by STRUCTURE analysis.**

| **Locus** | **Pop** | **P** | **A** | **H*_e_*** | **H*_o_*** | **F*_IS_*** |
| --- | --- | --- | --- | --- | --- | --- |
| **Lm2TG** |  |  |  |  |  |  |
|  | 1 | 1 | 2 | 0.13 | 0.04 | 0.66 |
|  | 2 | 1 | 4 | 0.25 | 0.08 | 0.68 |
|  | 3 | 1 | 5 | 0.67 | 0.08 | 0.89 |
|  | 4 | 1 | 5 | 0.37 | 0.14 | 0.62 |
| **Lm4TA** |  |  |  |  |  |  |
|  | 1 | 1 | 2 | 0.09 | 0.09 | -0.02 |
|  | 2 | 1 | 4 | 0.25 | 0.04 | 0.84 |
|  | 3 | 1 | 5 | 0.74 | 0.12 | 0.85 |
|  | 4 | 1 | 8 | 0.78 | 0.22 | 0.73 |
| **Li41-56 (B)** |  |  |  |  |  |  |
|  | 1 | 0 | 1 | 0 | 0 | 0 |
|  | 2 | 1 | 2 | 0.30 | 0 | 1 |
|  | 3 | 0 | 1 | 0 | 0 | 0 |
|  | 4 | 1 | 3 | 0.40 | 0 | 1 |
| **Li71-7 (R)** |  |  |  |  |  |  |
|  | 1 | 0 | 1 | 0 | 0 | 0 |
|  | 2 | 1 | 2 | 0.04 | 0 | 1 |
|  | 3 | 1 | 3 | 0.25 | 0.04 | 0.85 |
|  | 4 | 1 | 2 | 0.23 | 0.04 | 0.85 |
| **Li22-35 (E)** |  |  |  |  |  |  |
|  | 1 | 1 | 2 | 0.09 | 0 | 1 |
|  | 2 | 1 | 4 | 0.15 | 0.06 | 0.61 |
|  | 3 | 1 | 5 | 0.58 | 0.04 | 0.94 |
|  | 4 | 1 | 3 | 0.42 | 0.11 | 0.75 |
| **Li23-41 (F)** |  |  |  |  |  |  |
|  | 1 | 1 | 2 | 0.09 | 0 | 1 |
|  | 2 | 1 | 4 | 0.60 | 0.04 | 0.94 |
|  | 3 | 1 | 2 | 0.08 | 0 | 1 |
|  | 4 | 1 | 2 | 0.07 | 0 | 1 |
| **Li45-24 (G)** |  |  |  |  |  |  |
|  | 1 | 0 | 1 | 0 | 0 | 0 |
|  | 2 | 0 | 2 | 0.02 | 0.02 | 0 |
|  | 3 | 1 | 3 | 0.45 | 0.04 | 0.92 |
|  | 4 | 1 | 5 | 0.65 | 0.04 | 0.95 |
| **CS20** |  |  |  |  |  |  |
|  | 1 | 1 | 2 | 0.09 | 0 | 1 |
|  | 2 | 1 | 2 | 0.04 | 0 | 1 |
|  | 3 | 1 | 2 | 0.40 | 0 | 1 |
|  | 4 | 1 | 3 | 0.10 | 0.04 | 0.66 |
| **LIST7031** |  |  |  |  |  |  |
|  | 1 | 0 | 1 | 0 | 0 | 0 |
|  | 2 | 1 | 2 | 0.04 | 0 | 1 |
|  | 3 | 1 | 2 | 0.38 | 0.04 | 0.90 |
|  | 4 | 0 | 1 | 0 | 0 | 0 |
| **LIST7039** |  |  |  |  |  |  |
|  | 1 | 0 | 1 | 0 | 0 | 0 |
|  | 2 | 0 | 1 | 0 | 0 | 0 |
|  | 3 | 1 | 2 | 0.08 | 0 | 1 |
|  | 4 | 1 | 3 | 0.33 | 0.04 | 0.90 |
| **Li71-33 (P)** |  |  |  |  |  |  |
|  | 1 | 0 | 1 | 0 | 0 | 0 |
|  | 2 | 0 | 1 | 0 | 0 | 0 |
|  | 3 | 1 | 2 | 0.08 | 0 | 1 |
|  | 4 | 1 | 2 | 0.07 | 0 | 1 |
| **Li71-5/2 (Q)** |  |  |  |  |  |  |
|  | 1 | 0 | 1 | 0 | 0 | 0 |
|  | 2 | 1 | 2 | 0.08 | 0 | 1 |
|  | 3 | 1 | 3 | 0.22 | 0 | 1 |
|  | 4 | 0 | 1 | 0 | 0 | 0 |
| **TubCA** |  |  |  |  |  |  |
|  | 1 | 0 | 1 | 0 | 0 | 0 |
|  | 2 | 0 | 1 | 0 | 0 | 0 |
|  | 3 | 1 | 2 | 0.08 | 0 | 1 |
|  | 4 | 0 | 1 | 0 | 0 | 0 |

The MLMT data are based on the analysis 128 *L. infantum* strains in overall 14 microsatellite loci; marker Li46-67 (C) is monomorphic in all populations (data not shown). P, proportion of polymorphic loci; A, number of alleles; H*_e_*, Nei's unbiased expected heterozygosity; H*_o_*, observed heterozygosity; F*_is_*, inbreeding coefficient.
