# Supplementary material for: Genetic diversity and structure in Leishmania infantum populations from southeastern Europe revealed by microsatellite analysis
Source: Parasit Vectors. 2013 Dec 5;6:342. doi: 10.1186/1756-3305-6-342 (PMC4029556; doi:10.1186/1756-3305-6-342)
Supplement: Additional file 3: Table S2 — Descriptive statistics and comparison of FIS values between the sub-populations defined by STRUCTURE. [file 1756-3305-6-342-S3.doc]

**Additional File 3**

**Table S2. Descriptive statistics and comparison of F*_IS_* values between the sub-populations defined by STRUCTURE.**

| **Subpopulation** | **Origin** | **P** | **A** | **H*_e_*** | **H*_o_*** | **F*_IS_*** |
| --- | --- | --- | --- | --- | --- | --- |
| **3A** (7) | ES islands | 0.36 | 1.50 | 0.13 | 0.05 | 0.63 |
| **3B^1^** (14) | TR, HR, FR, ES, PT | 0.72 | 2.29 | 0.22 | 0.01 | 0.95 |
| **3B^2^** (5) | HR | 0.07 | 1.07 | 0.04 | 0.03 | 0.27 |
| **4A** (13) | TR, GR (MON-98) | 0.36 | 1.50 | 0.07 | 0.02 | 0.69 |
| **4B** (5) | AL, HR | 0.22 | 1.36 | 0.09 | 0.06 | 0.41 |
| **4C^1^** (4) | TR (MON-1), GR (MON-98) | 0.57 | 1.93 | 0.31 | 0.07 | 0.80 |
| **4C^2^** (6) | GR (MON-1) | 0.43 | 1.57 | 0.20 | 0.06 | 0.72 |

P, proportion of polymorphic loci; A, number of alleles; H*_e_*, Nei's unbiased expected heterozygosity; H*_o_*, observed heterozygosity; F*_is_*, inbreeding coefficient.
