# Supplementary material for: Genetic diversity and structure in Leishmania infantum populations from southeastern Europe revealed by microsatellite analysis
Source: Parasit Vectors. 2013 Dec 5;6:342. doi: 10.1186/1756-3305-6-342 (PMC4029556; doi:10.1186/1756-3305-6-342)
Supplement: Additional file 4: Table S3 — FST values and corresponding p-values for the sub-populations identified by STRUCTURE re-analysis. [file 1756-3305-6-342-S4.doc]

**Additional File 4**

**Table S3. F_ST_ values and corresponding *p*-values for the sub-populations identified by STRUCTURE re-analysis.**

|  | **1** | **2** | **3A** | **3B^1^** | **3B^2^** | **4A** | **4B** | **4C^1^** | **4C^2^** |
| --- | --- | --- | --- | --- | --- | --- | --- | --- | --- |
| **1** | 0 | 0.655 | 0.866 | 0.690 | 0.879 | 0.866 | 0.837 | 0.775 | 0.690 |
| **2** | <0.001 | 0 | 0.739 | 0.623 | 0.604 | 0.619 | 0.553 | 0.504 | 0.540 |
| **3A** | <0.001 | <0.001 | 0 | 0.452 | 0.787 | 0.813 | 0.765 | 0.575 | 0.633 |
| **3B^1^** | <0.001 | <0.001 | <0.001 | 0 | 0.475 | 0.629 | 0.539 | 0.382 | 0.448 |
| **3B^2^** | <0.001 | <0.001 | <0.001 | <0.001 | 0 | 0.821 | 0.812 | 0.490 | 0.601 |
| **4A** | <0.001 | <0.001 | <0.001 | <0.001 | <0.001 | 0 | 0.718 | 0.381 | 0.587 |
| **4B** | <0.001 | <0.001 | <0.001 | <0.001 | <0.006 | <0.001 | 0 | 0.433 | 0.528 |
| **4C^1^** | <0.001 | <0.001 | <0.006 | <0.001 | <0.006 | <0.001 | <0.001 | 0 | 0.249 |
| **4C^2^** | <0.001 | <0.001 | <0.001 | <0.001 | <0.006 | <0.001 | <0.001 | <0.001 | 0 |

F_ST_, pairwise Wright’s fixation index (upper right triangle); *p*-values, confidence test (lower left triangle).
